# Diverse reference genomes detect variants in the US winter wheat

**DOI:** 10.1002/tpg2.70160

**Published:** 2025-12-30

**Authors:** Kyle Parker, Zhen Wang, Yahya Rauf, Liangliang Gao, Jesse Poland, Jackie Rudd, Amir Ibrahim, Shuyu Liu

**Affiliations:** ^1^ Department of Soil and Crop Sciences Texas A&M University College Station Texas USA; ^2^ Department of Agronomy Kansas State University Manhattan Kansas USA; ^3^ Department of Plant Science King Abdullah University of Science and Technology Thuwal Saudi Arabia; ^4^ Texas A&M AgriLife Research Amarillo Texas USA; ^5^ Texas A&M AgriLife Research College Station Texas USA

## Abstract

The first hexaploid bread wheat reference genome from Chinese Spring was released in 2018 by the International Wheat Genome Sequencing Consortium and is considered as the industry standard reference. To explore the effects of different reference genomes on variant discovery, 29 hexaploid bread wheat (*Triticum aestivum* L.) cultivars from the Southern Great Plains of the United States with varying whole genome sequencing depth were aligned to three reference genomes. The reference genomes varied in evolutionary similarity to the cohort of the germplasm analyzed: (1) Jagger, a Kansas State University cultivar, a reference with high similarity, (2) Chinese Spring, a Chinese landrace, the current industry standard, (3) Durum‐*tauschii*, an “in silico” hybridization of the reference genomes of tetraploid durum wheat and wild *Aegilops tauschii*, for an unrepresentative reference. The Jagger reference genome retained more informative variants after filtering. Synteny regions and large introgressions were identified by read alignment coverage, using diverse reference genomes to identify lines containing *Aegilops ventricosa* (2N^v^S), 1AL:1RS and 1BL:1RS (*Secale cereale*) rye translocations, and a 2.8 Mb contig from TAM 112 known to contain a haplotype harboring greenbug [*Schizaphis graminum* (Rondani)] resistance gene *Gb3*. The choice of reference genome is important, but any single reference can induce bias. This study with US Southern Great Plains germplasm demonstrated the importance of multiple reference genomes to capture genetic diversity.

## Plain Language Summary

As we continue in the genomics era, more reference genomes are available for researchers. This study seeks to explore the effects of using alternative reference genomes with varying genetic similarity to a cohort of samples for identifying unique genetic variations. By evaluating the different effects of multiple reference genomes, we identified the variations that occur when discovering genetic differences. We observed that a more closely related reference genome can decrease the number of errors when aligning a whole genome sequence. Similarly, if there are unique chromosomal portions under studying genomes, those can be only identified if they are in fact present in the reference genome. By using a diverse set of reference genomes, we can identify the unique genetic differences in the populations. This study demonstrates the importance of using the best reference genome to identify if a sample has a unique region of interest.

**Abbreviations**
BAM: binary alignment map
CS: Chinese Spring
INDEL: insertion/deletion
JG: Jagger
MAF: minor allele frequency
MAF3: minor allele frequency of 3%
MAF5: minor allele frequency of 5%
MQ: mapping quality
NCBI: National Center for Biotechnology Information
PAV: presence–absence variation
SNP: single‐nucleotide polymorphism
SO: sequence ontology
VCF: variant calling format
WGS: whole genome sequencing

## 1 INTRODUCTION

In the genomics era, one indispensable tool for any researcher conducting a genetic study is the availability of a reference genome for their organism. With the advent of genotyping‐by‐sequencing technologies, researchers have been able to generate higher quantity and cheaper short‐read sequencing data, and the alignment to a reference genome has become an integral part during variant discovery (Elshire et al., 2011). Short‐read sequencing data can be likened to individual pieces of a jigsaw puzzle. Without a reference genome as a guiding picture, assembling these pieces can be challenging. However, by utilizing a reference genome, the process of aligning genomic data becomes analogous to placing puzzle pieces in their respective positions based on the provided picture.

The first reference genome of a multicellular organism, consisting of 97 Mb and revealing over 19,000 genes, came from the nematode *Caenorhabditis elegans* in 1998 (The C. elegans Sequencing Consortium, 1998). The draft of the human (*Homo sapiens*) genome was published in 2001 (Wolfsberg et al., 2001), and the first near‐complete assembly in 2004 with the genome size of 3.08 Gb (International Human Genome Sequencing Consortium, 2004). Among crops, the rice (*Oryza sativa*) genome was published in 2002 (420 Mb) (Goff et al., 2002). While the first bread wheat (*Triticum aestivum* L.) genome publishing was developmentally behind, partially due to its complexity and large, highly repetitive hexaploid genome of 16 Gb. Hexaploid wheat had its first reference genome draft published in 2014 (International Wheat Genome Sequencing Consortium, 2014), and its complete genome reference was published in 2018 (The International Wheat Genome Sequencing Consortium, 2018), which used Chinese Spring (CS), the standard of wheat cytogenetic research. Most recently, in 2021, the wheat reference genome was updated to the latest version 2.1 (Zhu et al., 2021).

With the capability of some sequencing platforms, like PacBio to capture long sequencing reads up to ∼25 kb at high fidelity (Hon et al., 2020), reference‐grade assembles can be created easily with most available bioinformatics computational resources. Now, with the backbone references published for many crops, it raises the question: Does the standard reference most accurately represent the materials being analyzed? CS is a Chinese white wheat cultivar that was selected and used by the International Wheat Genome Sequencing Consortium for the first wheat genome assembly, due to its history in cytogenetics work (Liu et al., 2018). However, research is lacking to compare the effects of utilizing this standard reference and other more recent references when analyzing modern North American Great Plains wheat germplasm, and its impact on variant discovery.

Research studies in mice revealed that even between two inbred mice, the sequence between the reference and the aligned inbred could be as different as between humans and chimpanzees at specific genomic regions (Lilue et al., 2018). This can make it hard to know if a divergent read is misaligned or if it represents real allelic diversity. Similarly, researchers found many high‐confidence heterozygous calls, which should be homozygous genotypes, indicating the potential existence of novel genes, copy number variations, or other variants that are not represented in the reference, causing an error in read mapping (Keane et al., 2011).

Synthetic hexaploids have been used for many years as a source of novel genetic variation to improve modern cultivars (A. Li et al., 2018; Morgounov et al., 2018; Mujeeb‐Kazi et al., 2009, 2008). The winter wheat germplasm in the US Southern Great Plains has benefited from the introduction of superior traits by crossing with exotic materials such as synthetic hexaploid wheats (Cooper et al., 2013, 2012; Crespo‐Herrera et al., 2019; Dreisigacker et al., 2008). These synthetic hexaploid wheats recreate the hybridization events between the AB genomes donor, durum wheat (*Triticum turgidum* L. ssp. *durum*), and the D genome donor goat grass (*Aegilops tauschii*). These synthetic hexaploids are thus a composite of modern tetraploid durum wheats and the wild *A. tauschii* accessions. A popular cultivar TAM 112 has an alien introgression from *A. tauschii* TA2460. Synthetic wheat Largo in TAM 112 pedigree contains green bug resistance gene *Gb3* from PI268210 (TA1618), as well as a chromosomal translocation (1AL:1RS) from rye (*Secale cereale*) (Rudd et al., 2014), and many other popular lines of the Southern plains share similar histories of introgression.

Efforts were taken to capture the diversity in cultivated wheat by creating a pan‐genome utilizing 18 diverse bread wheat cultivars, including CS and Jagger (JG) (Montenegro et al., 2017). By utilizing an iterative mapping approach, researchers were able to identify the core and pan‐genome comprised of ∼89,745 and ∼140,500 genes, respectively (Montenegro et al., 2017). Based on these results, Bayer et al. (2022) developed a graphical pan‐genome as well as an online tool to visualize the genomic haplotypes (Wheat Panache). This demonstrated an increase of ∼1.2 Gb of the graphical pan‐genome compared to the base reference of solely CS. The CS reference was found to have the most unique genes in the panel (∼2216 genes) (Bayer et al., 2022), most likely because of its far evolutionary distance from the modern cultivars.

Synteny, or the conservation of relative gene order among different species (Lovell et al., 2022), can be an informative way to understand the effects of evolutionary distance on the genome. Synteny can be commonly observed using gene collinearity and orthogroups. Utilizing orthogroups instead of solely gene colinearity can be especially helpful in polyploid organisms, reducing the noise caused by paralog and other gene duplication events (Walden & Schranz, 2023).

This study seeks to understand the effects of using alternative reference genomes, with varying evolutionary distance, on variant discovery from a collection of Southern Great Plains bread wheat germplasm. The Southern Great Plains, generally made up of Colorado, Kansas, Oklahoma, and Texas states, account for ∼43% of the total wheat acres planted in the United States during 2023 (USDA‐NASS, 2024). The cultivars evaluated in this study are important parents in many of the Southern Great Plains breeding programs. Evaluating the effects of reference bias and how to leverage alignment coverage to identify introgressions in this population will provide breeders a better understanding of the genomic tools available and how to maximize their utility.

## 2 MATERIALS AND METHODS

### 2.1 Plant materials and variant calling

Twenty‐nine hexaploid wheat (*T. aestivum* L.) cultivars, or elite lines, adapted to the Southern Great Plains region of the North America were sequenced previously as part of the National Center for Biotechnology Information (NCBI) BioProject PRJNA694980 (Gaurav et al., 2022). Whole genome sequencing (WGS) was performed with varying coverages ranging from 5× to 20× (Table S1). CS short reads were downloaded from NCBI BioProject PRJNA393343 (The International Wheat Genome Sequencing Consortium, 2018), while JG was downloaded from NCBI BioProject PRJNA544491 (Walkowiak et al., 2020). Raw paired‐end FASTQ files were downloaded from the NCBI using SRAtools (https://github.com/ncbi/sra-tools). FASTQ files were aligned to three different reference genomes using the Burrows–Wheeler Aligner “BWA‐MEM2” with default settings (Vasimuddin et al., 2019). Sequence alignment map files were sorted by position and converted to binary alignment map (BAM) files. Sorted BAMs had non‐primary alignment reads removed before using the function “MarkedDuplicates” of Picard Tools (http://broadinstitute.github.io/picard) to designate the duplicate reads. Finally, unmapped reads were removed from the BAM file using “SAMtools view ‐F4” (H. Li et al., 2009). This process was repeated for each of the three reference genomes including Chinese Spring v2.1 (CS), JG (Walkowiak et al., 2020), and a custom in “silico” hybridization of a synthetic hexaploid wheat by the concatenation of *T. turgidum* L. ssp. *durum* reference Svevo V1 (Maccaferri et al., 2019) and *A. tauschii* reference AL8/78 V5.0 (L. Wang et al., 2021) referred hereafter as DT for Durum*‐tauschii*. “Samtools stats” was used to generate alignment statistics for each of the BAM files before and after alignment preprocessing. Likewise, “Samtools coverage” was used to generate coverage statistics on processed BAM files. BCFtools (H. Li, 2011) “mpileup” and “call” were used to call single‐nucleotide polymorphism (SNP) and insertions/deletions (INDELs). The identified variants were then filtered at 3% and 5% minor allele frequency (MAF), maximum missing value of 10% per locus, and maximum heterozygosity of 10% using BCFtools. This variant calling workflow can be duplicated using NexGenSnake (Parker, 2024).

Due to the varied levels of sequence information available for each line, a proportion was used instead of the raw read number or base number for the alignment statistical analysis. The percentage of duplicated read analysis was conducted on the post‐filtered BAM files on account of data availability.

Statistical analysis was performed using JMP Pro Version 17. Due to the non‐normality of the data, nonparametric Steel–Dwass comparisons for all pairs of tests were conducted to find out significant differences between reference groups. Outliers tended to be the same lines across all references reflecting the individual differences between the genotypes, so they were retained during analysis.

### 2.2 Synteny

Collinearity was calculated by “TBtools” (Chen et al., 2020) and visualized using “MCscanX” (Y. Wang et al., 2012). This analysis was limited to pairwise analysis. All annotation files were downloaded before March 31, 2023. The synthetic annotation file was created by concatenating the *T. turgidum* and the *A. tauschii* annotation files and formatting to match gff3 format. Across all genomes, GENESPACE (Lovell et al., 2022) was employed using default settings to run synteny analysis by ortholog identification and visualization of synteny.

### 2.3 Introgression identification

Coverage data collected from “samtools coverage” on processed BAM files were used in conjunction with historical marker data collected by the United States Department of Agriculture (USDA) Central Small Grains Genotyping Laboratory at Manhattan, KS, for validation. Presence–absence variation (PAV) of the *Aegilops ventricosa* 2N^v^S chromosomal segment present on chromosome 2A was utilized for the comparison of coverage between CS and JG on chromosome 2A. For the identification of rye chromosome introgression on both 1A and 1B, coverage data were analyzed for chromosomes 1A and 1B. To validate the introgression, lines were then realigned to a JG reference in combination with chromosome 1R of rye (*S. cereale*) reference Weining V1 (G. Li et al., 2021). To generate coverage depth graphs, the number of mapped reads per megabase were binned and visualized using a custom Python script.

To test for PAV of the *Gb3* haplotype, a contig containing TAM 112′s distal end of 7D (Azhaguvel et al., 2012; Rudd et al., 2014) and otherwise unmapped reads after alignment to CS were assembled using “MaSuRCA” V4.0.5 (Zimin et al., 2013). The assembled contigs were then aligned again to CS with “minimap2” (H. Li, 2018). Contigs that fell within the distal end of chromosome 7D (597–609 Mb) and had a mapping quality (MQ) of greater than 20 were combined to create the tamGB3 contig. While aligning reads from the WGS, the tamGB3 contig was included with the Chinese Spring V2.1 reference genome to allow for relative comparisons between CS 7D and the tamGB3 contig in alignment.

The three lines that contained *Gb3* showed three unique regions on the contig where read coverage was high in the *Gb3*‐positive lines and low in the rest of the lines. Region 1 covered 12,509 bp of the tamGB3 contig, which translated to a 56,758 region in CS 7D. Region 2 and Region 3 were larger, covering 62,528 and 39,968 bp in tamGB3, corresponding to 261,554 bp and 181.723 bp in CS 7D, respectively. The tamGB3 contig was 2.8 Mb, corresponding to a 12 Mb region in CS 7D. The tamGB3 contig contained ∼9.484 Mb in gaps when compared with CS 7D.

### 2.4 VCF stats

VCF (variant calling format) files were annotated by “snpEff” (Baets et al., 2012) with default settings. JG reference used a built‐in database “*Triticum_aestivum*_jagger,” while CS had a custom‐loaded database using the annotations from the most recent Chinese Spring V2.1 annotations. DT likewise used a custom‐loaded database from the annotation set of the Durum*‐tauschii* reference. Metrics that were generated after running annotations on the VCF files were compared.

Allele/variant counts were taken from the output of the snpEff, and the percentage of alleles was calculated by taking the specific allele type, that is, the number of heterozygous, missing, and reference alleles, and dividing it by the total number of variants. Raw VCF files were used for evaluating the proportion of heterozygous and missing variants, since the filtered variant calling files were already filtered for missing percentage and for heterozygosity at each locus. For the percentage of reference alleles, both the filtered and raw VCFs were analyzed. The percentage of reference alleles did not follow a normal distribution; therefore, Wilcox paired rank test was implemented for the comparisons. The non‐normality of the results reflected the diversity in the germplasm as well as the varied sequencing depth.

### 2.5 Variant effects

Ensembl variant effects were calculated by utilizing annotation files to predict the effect of the variant change. Each variant was evaluated against the annotation files to find the sequence ontology (SO) term that can be applied to the variant. Then, in turn, each SO term corresponds to an impact rating depending on the predicted effects of the variant from the reference sequence (Ensembl Variation—Calculated variant consequences). Following similar methods as outlined by Torkamaneh et al. (2018), “high” effect variants correspond to SO terms of stop gained/lost, frameshifts, and splice donor/acceptor variants, and so forth. These effects tend to impact protein function. Moderate effects are in‐frame insertions/deletions, missense, or nondisruptive variations that might change protein effectiveness, while “low” effects are incomplete terminal codon variant, splice region variants, synonymous, or start/stop retained variants, which are unlikely to affect protein behavior. “Modifier” variant effects occupied the rest, such as intron variants, upstream/downstream, noncoding transcript variant, and intergenic variants, and these variants are typically noncoding.

### 2.6 Diversity

Phylogenetic trees and allelic diversity were calculated in TASSEL (Bradbury et al., 2007). The phylogenetic trees were calculated utilizing neighbor joining and visualized in iTOL (Letunic & Bork, 2021). Allelic diversity was measured as *θπ* (Pi) and visualized through a custom *R* script using the R package *ggplot2* (Wickham, 2016), graphing the per‐base Pi value.

## 3 RESULTS

### 3.1 Reference comparisons

The 29 WGS lines and JG are elite hexaploid wheat cultivars released for the North American, Southern Great Plains, with many varieties released from Oklahoma, Kansas, and Texas (Table S1, Figure S1). Three different references were compared (1) the industry standard, CS, which is a Chinese white spring wheat, (2) a representative cultivar of the Southern Great Plains, JG, a hard red winter wheat variety released by Kansas State University, and (3) a nonrepresentative synthetic wheat reference, DT, comprised of an Italian durum wheat and a wild *A. tauschii* accession.

The different reference genomes have differing lengths of chromosomes; differing by up to 44 Mb on chromosome 5A between CS/JG and DT, and as little as 17 kb on chromosome 6D between CS and DT (Table S2). The average chromosomal difference was much higher between the DT and the CS/JG references, with an average length of ∼15 Mb, while the average difference between the JG and CS was ∼7 Mb per chromosome, ranging from 36 Mb on chromosome 6D and only 39 kb on chromosome 5A. Between JG and CS references, the largest differences were observed on the D genome followed by the B and A genomes, each with an average difference of 9.6, 6.7, and 4.7 Mb, respectively. The relative GC percentage was conserved across all references of 46%, except for the *A. tauschii* reference for the D genome of DT, which had a GC content of 46.5%.

### 3.2 Synteny

After running pairwise collinearity analysis with TBtools and MCScanX, the JG/CS references had the largest number of colinear genes 182,438 (73.77%) out of 247,291 total genes (Table 1). CS/DT shared the highest percentage of collinearity with 162,704 (75.42%) collinear genes out of 215,730 genes. However, JG/DT had the lowest percentage of collinearity, with 66.88%. Of the pairwise comparisons, JG/DT contained the most unique genes, with 52,677 genes from JG and 29,865 genes from DT. JG had approximately three unique genes for every unique gene from CS (48,718/16,135). However, CS/DT contributed roughly a similar number of unique genes 22,647 and 30,379, respectively.

**TABLE 1:** Reference genome collinearity.

Collinearity			
Pairwise comparison	JG‐CS	CS‐DT	JG‐DT
Collinear genes	182,438	162,704	166,661
Unique genes	48,718 JG | 16,135 CS	22,647 CS | DT 30,379	52,677 JG | DT 29,865
Total unique genes	64,853	53,026	82,542
Total genes	247,291	215,730	249,203
% of collinear genes	73.77	75.42	66.88

*Note*: Chinese Spring (CS), Synthetic (DT), Jagger (JG) reference, pairwise comparisons of gene collinearity between three references genomes. Genes are identified from the respective published annotation files for each reference. Gene collinearity is a function of conserved gene order.

**FIGURE 1:**
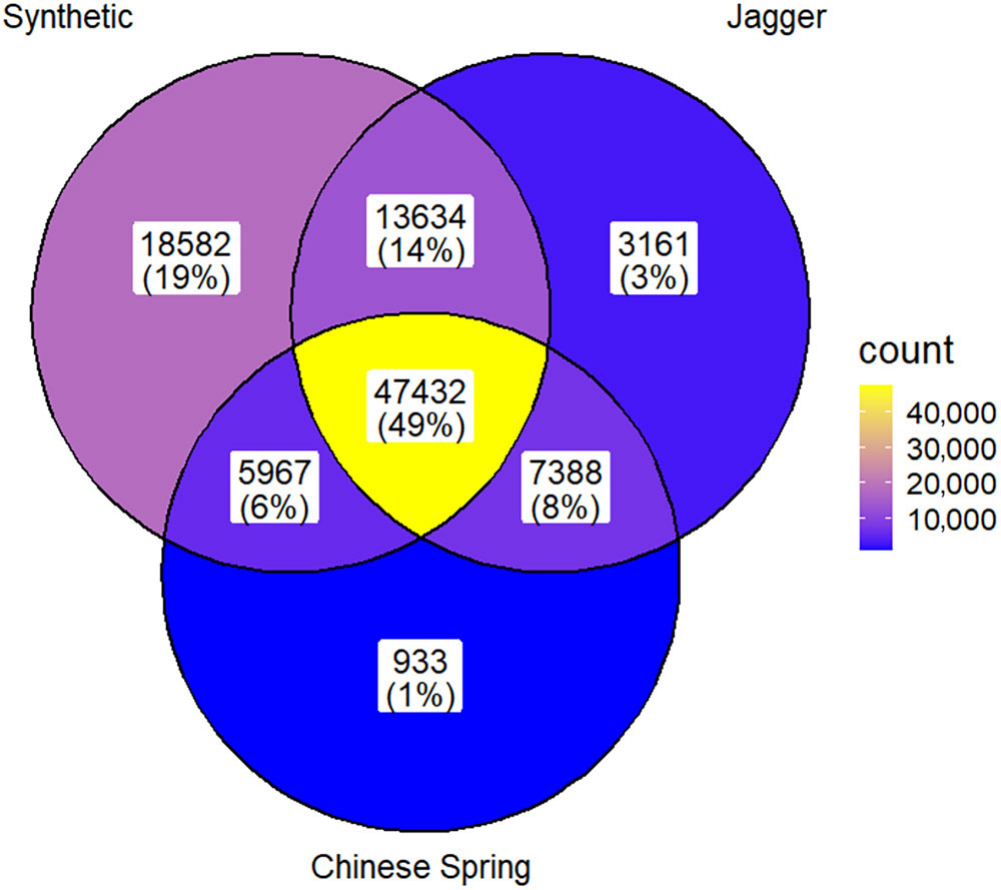
Venn diagram showing orthogroup distribution between three reference genomes. The lighter color (yellow) indicates higher number of orthogroups, while the cooler (blue) indicates a smaller number of orthogroups.

The orthogroup analysis performed by GENESPACE, utilizing reference genome annotation files, revealed shared orthologous genes in each of the references, allowing the direct comparison between all three references. The DT reference contained the most total orthogroups (85,615), followed by JG (71,615) and CS (61,719) (Table 2). The JG/DT references shared the highest number of orthogroups (61,066). CS and JG shared the highest percentage of shared orthogroups 88.82% (Figure 1).

**TABLE 2:** Shared reference orthogroups.

Orthogroups shared			
	CS	DT	JG
**CS**	**61,719**	**53,398**	**54,819**
	100%	86.52%	88.82%
**DT**	**53,398**	**85,615**	**61,066**
	62.37%	100%	71.33%
**JG**	**54,819**	**61,066**	**71,615**
	76.55%	74.56%	100%

*Note*: Chinese Spring (CS), Jagger (JG), and Synthetic (DT) shared orthogroups from GENESPACE. Values in bold are the shared number of orthogroups between the reference on the *x* and *y* axis. Percentages are calculated by using the references on the *y*‐axis as the denominator and the reference displayed on the *x*‐axis as the numerator.

**FIGURE 2:**
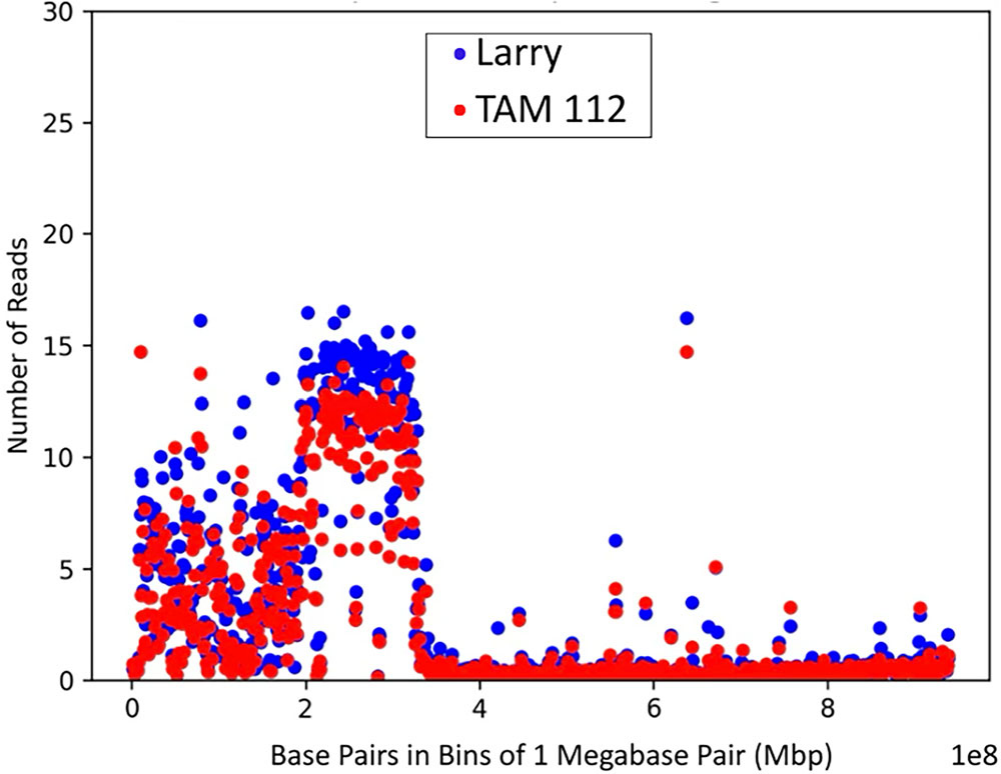
1R alignment. Alignment coverage represented as the number of reads aligned per megabase pair (1 Mb) bin to rye chromosome 1. Larry, a cultivar with 1BL:1RS translocation, is in blue, while TAM 112, a cultivar with the 1AL:1RS translocation, is in red.

CS maintained the highest percentage of overall genes assigned to orthogroups (95.5%) and had the least amount of reference‐specific groups (933) (Figure 2). Inversely, the DT reference had the most genes not assigned to orthogroups and the most amount of reference‐specific orthogroups (Table 3).

**TABLE 3:** Reference specific orthogroups.

	CS	DT	JG
Total genes	103,787	236,127	160,615
Genes in orthogroups	99,145	188,884	141,740
Number of unassigned genes	4642	47,243	18,875
Percentage of genes in orthogroups	95.50%	80%	88.20%
Percentage of unassigned genes	4.50%	20%	11.80%
Total orthogroups in each reference	61,719	85,615	71,615
Percentage of orthogroups containing reference	63.60%	88.20%	73.80%
Reference‐specific orthogroups	933	18,582	3161
Genes in reference‐specific orthogroups	2716	80,130	20,548
Genes in reference‐specific orthogroups	2.60%	33.90%	12.80%
Overall metrics			
Overall genes assigned to an orthogroup	85.90%		
Total orthogroups	97,097		
Orthogroups present universally	47,432		
Genes in reference‐specific orthogroups	20.70%		

*Note*: Orthogroup metrics for each of the three references: Chinese Spring (CS), Durum‐*tauschii* (synthetics, DT), and Jagger (JG) from GENESPACE.

Across references, there were 97,097 total orthogroups, representing 85.9% of the annotated genes. Of the total orthogroups, 48.8% were conserved across all references. Of the 85.9% of the annotated genes in orthogroups, a fifth of them (20.7%) represented reference‐specific diversity and grouped into reference‐specific orthogroups.

### 3.3 Alignments

Statistically, DT (0.87%) had significantly more supplementary aligned reads when compared to CS (0.61%) and JG (0.60%) references, while CS and JG were not significantly different from each other (Table S3). However, the percentage of unmapped reads did not significantly differ between the different reference genomes (Figure S2). Using the filtered alignment files, we observed no significant differences in average MQ (∼35) and the read duplication percentage (6%). DT was found to have a significantly lower percentage of properly paired reads to CS, with 95.46% versus 96.17.% (Table S3).

Similarly, DT (1.16%) had a significant increase in reads paired on multiple chromosomes than JG/CS (0.90% and 0.90%). The JG reference showed significantly lower reads with a MQ score of 0 (12.92%), as well as the lowest average base error rate (0.009). CS and DT did not have a significant difference for reads with an MQ of 0 (15.84% and 16.38%), but CS and JG did have a significantly lower average base error rate than DT, 0.01 and 0.013, respectively.

### 3.4 Coverage

When looking at chromosome coverage in terms of covered bases per chromosome, the A genome had no significant difference among the three references (∼86% coverage), except for chromosome 7A, where JG demonstrated significantly higher coverage than DT, while both were statistically similar to CS (Table S4, Figure S3). For most of the D genome, CS and JG had better coverage than DT, except on chromosome 2D where CS (88.80%) was greater than both JG (84.10%) and DT (85.04%). JG and DT statistically had no difference on chromosome 2D. In the B genome overall, JG (86.82%) showed significantly higher coverage than CS (85.24%) and DT (84.70%), which were not significantly different. Chromosomes 1B, 2B, 6B, and 7B showed significant differences in coverage among the three references. For both 1B and 7B chromosomes, JG had the highest coverage with 85.59% and 88.06%, respectively, while DT and CS were statistically similar. Chromosome 2B showed a significant difference for JG (85.68%), having higher coverage than DT (83.24%); however, CS (83.83%) was not significantly different from either DT or JG. For 6B, both JG (87.16%) and DT (85.29%) had similar coverage, and CS (82.97%) had significantly lower coverage.

### 3.5 Alien chromosome fragment identification

Coverage analysis was used to identify the PAV of the *A. ventricosa* 2N^v^S chromosomal segment present on chromosome 2A. Lines with a significant increase in coverage on chromosome 2A from alignment from CS to JG were predicted to harbor the 2N^v^S segment. Historical marker data collected by the USDA Central Small Grains Genotyping Laboratory at Manhattan, KS, and previous publications were used as validation (Gao et al., 2021; Mustahsan, 2022; Rudd et al., 2019) (Table S5). Lines without available validation data were then “predicted” based on coverage results.

**FIGURE 3:**
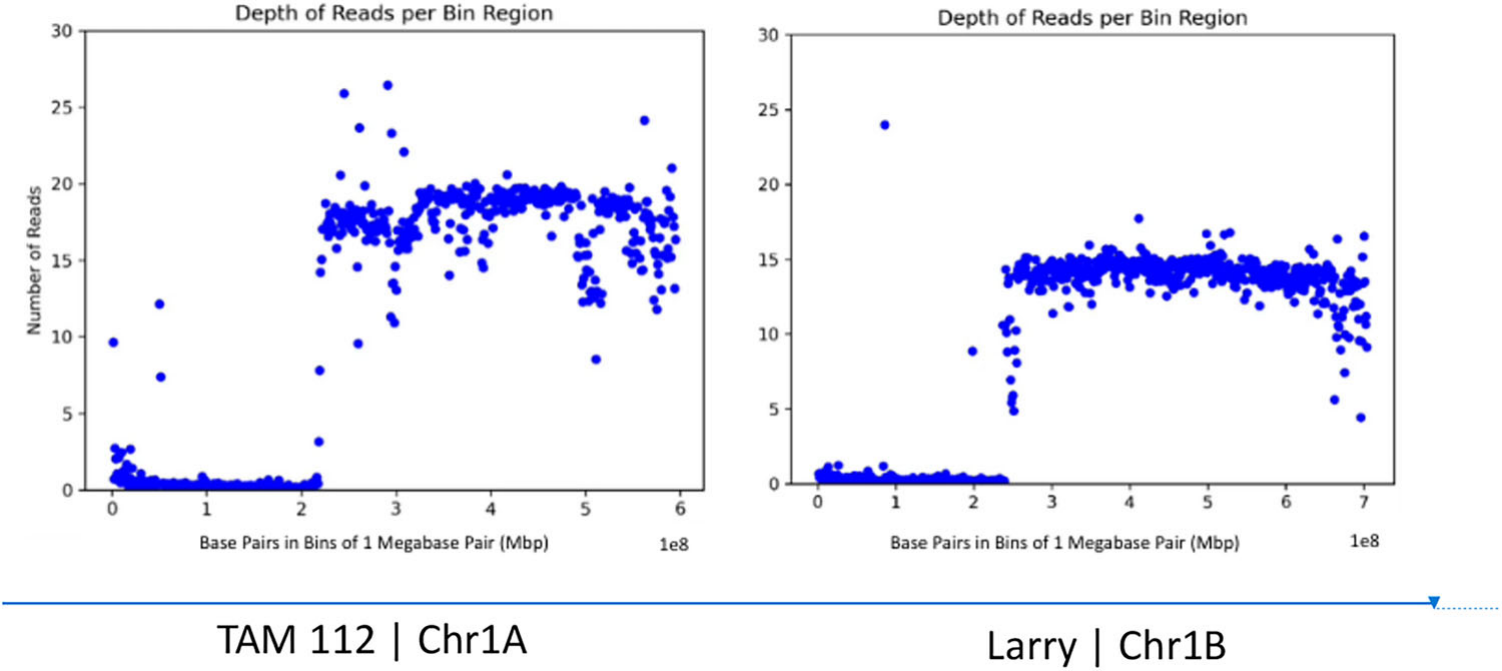
Alignment of 1AL:1RS and 1BL:1RS translocations. (Left) Alignment of TAM 112, which contains 1AL:1RS translocation to Chinese Spring chromosome 1A. (Right) Alignment of Larry, which contains 1BL:1RS translocation to Chinese Spring chromosome 1B.

PAV of the 1R rye translocation was initially observed in coverage analysis where for either chromosome 1A for the 1AL:1RS translocation, or 1B for the 1BL:1RS translocation, the chromosome coverage was reduced by ∼23% from the line's average (Table S6). Likewise, a drop in read depth was observed on the chromosomal regions that contained the rye translocation in comparison to the average read depth on chromosomal regions without the introgression (Figure 2). Lines that did not contain the translocation showed very low coverage of alignment to the 1R chromosome (<2%) and very low average read depth (<1). Alignment to a composite reference including the 1R chromosome validated the PAV of the translocation, resulting in ∼30% coverage of the 1R chromosome for lines with the translocation. Both 1B and 1A translocations appeared to contain the same region of the 1R chromosome (Figures 2 and 3). Lines that could not be validated by historical marker data were denoted as “predicted.”

PAV of *Gb3* resulted in only ∼10% coverage change between the tamGB3 contig and CS 7D chromosome (Table S7). Lines without *Gb3* observed a ∼30% drop in coverage when compared to the standard CS 7D. The three lines that contained *Gb3* showed three unique regions on the contig where read coverage was high in the *Gb3*‐positive lines and low in the rest of the lines (Figure S4, Table 4).

**TABLE 4:** Contig of tamGB3.

	tamGB3			Chinese Spring Chr 7D			
	Length (bp)	Start	End	Start	End	Length (bp)	Gaps (bp)
tamGB3	2,800,262	1	2,800,262	597,594,329	609,618,385	12,024,056	9,484,375
Region 1	12,509	483,735	496,244	599,644,736	599,701,494	56,758	45,368
Region 2	62,528	1,507,758	1,570,286	604,381,862	604,643,416	261,554	205,897
Region 3	39,968	2,621,288	2,661,256	608,922,872	609,104,595	181,723	145,529

*Note*: Length of each contig region is given in base pairs (bp). The contig tamGB3 is compared to the Chinese Spring chromosome 7D. (left) designates the positioning in relation to the tamGB3 contig, while (right) designates the relative location on Chinese Spring chromosome 7D. The gaps are the base pair length on Chinese Spring 7D that was not covered completely in the tamGB3 contig.

### 3.6 Variants discovered

**FIGURE 4:**
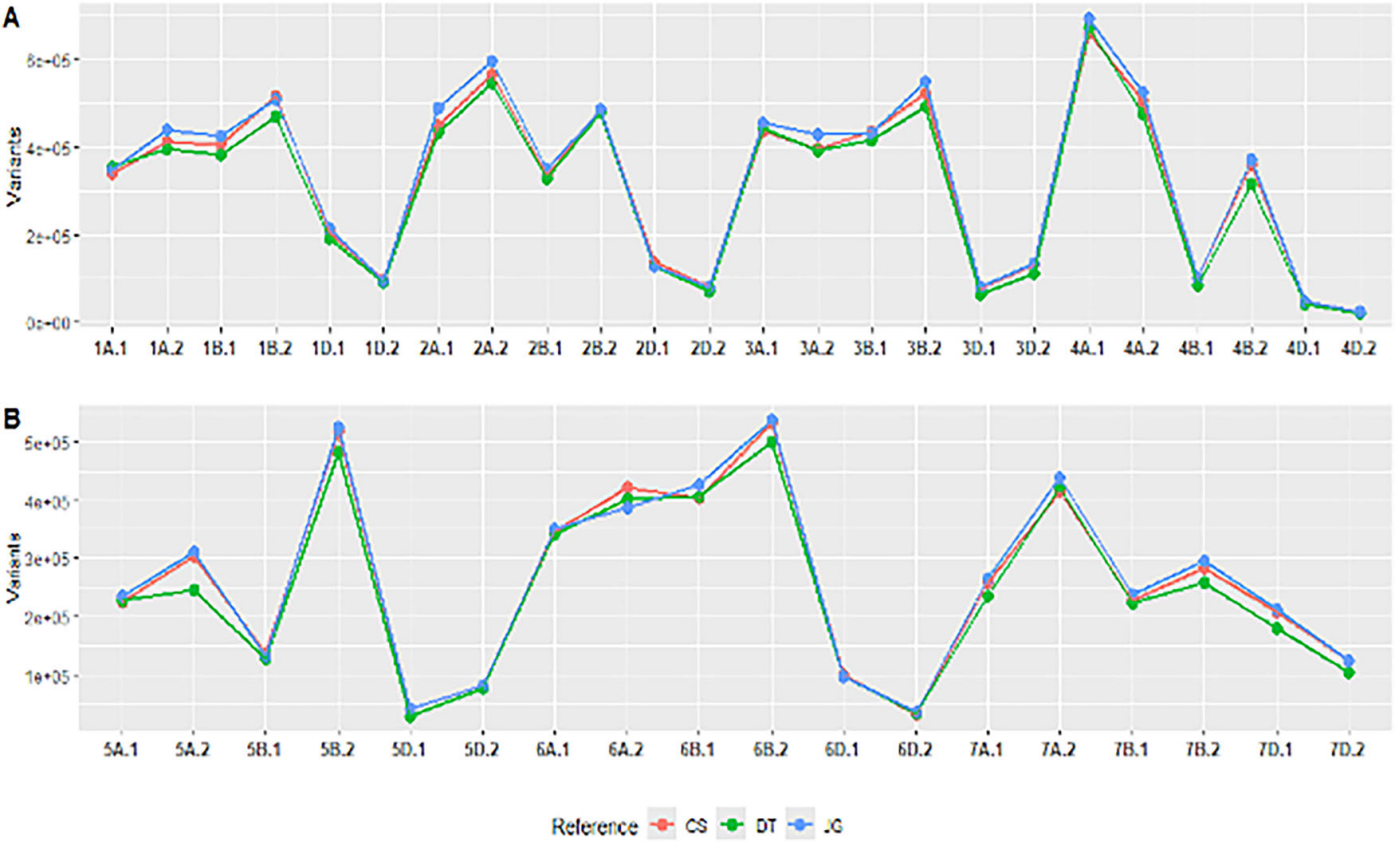
Variants per chromosome segment. (A) Chromosomes 1A to 4D and (B) chromosomes 5A to 7D for plots of variants discovered per chromosomal segment, designated by 0.1 for the short arm of the chromosome up until the centromere (short arm), and 0.2 is the long arm of the chromosome (long arm). Using filtered data with a minor allele frequency of 5%, and reference genomes Chinese Spring (CS) (red), Durum‐*t* *auschii* (DT) (green), and Jagger (JG) (blue).

Variant discovery varied among the three reference genomes. For unfiltered data, DT generated the most variants (275,783,108), followed by CS (222,385,125) and JG (193,848,321) (Table 5). The B genome had the highest number of variants followed by the A and D genomes, which is consistent with previous research (Bernardo et al., 2020; S. Wang et al., 2014; Z. Wang et al., 2022). When filtering for less than 10% missing, 3% MAF, and 10% maximum heterozygosity, the three references revealed very similar numbers of markers, with DT maintaining the most (31,508,758) followed by JG and then CS at 30,913,942 and 30,415,536, respectively. Then, finally filtering for less than 10% missing, 5% MAF, and 10% maximum heterozygosity, the results were reversed from the raw results, with JG (12,739,519) having a higher number of variants followed by CS (12,424,318) and DT (11,792,943). Since the physical locations of markers cannot be compared directly across different references, a binned approach was taken to observe the number of variants per chromosomal arm. Following the centromeric regions reported by Su et al. (2019), one can observe that the relative quantity of filtered variants per chromosome arm is conserved (Figure 4). Except for a few chromosome segments where a more significant difference was observed, like the distal end of 5A, DT discovered less variants than CS and JG.

**TABLE 5:** Variant Type Discovered.

	Raw			Filtered MAF 5%		
Type	JG	DT	CS	JG	DT	CS
SNP	186,101,191	263,644,119	213,655,396	11,831,581	10,888,422	11,509,063
INS	2,625,087	4,584,859	3,788,665	413,220	382,208	399,858
DEL	5,122,043	7,554,130	4,941,064	494,718	522,313	515,397
INDELs	7,747,130	12,138,989	8,729,729	907,938	904,521	915,255
Total	193,848,321	275,783,108	222,385,125	12,739,519	11,792,943	12,424,318

*Note*: Number of variants discovered for each of the three references Jagger (JG), Durum‐*tauschii* (DT), and Chinese Spring (CS). Single‐nucleotide polymorphism (SNP), insertion (INS), deletion (DEL), and insertions and deletions (INDELs). Where “raw” designated the unfiltered variant file, and filtered minor allele frequency (MAF) 5% is after filtering for minor allele frequency to be greater than 5%.

Overall, variants were subdivided into INDELs and SNPs. The SNPs followed the same pattern as the overall variants, with DT calling the most unfiltered SNPs (263,644,119) followed by CS (213,655,396) and JG (186,101,191). After filtering for minor allele frequency of 5% (MAF5), JG (11,831,581) retained the most, followed by CS (11,509,063) and DT (10,888,422). The total number of discovered INDELs was highest from DT before filtering (12,138,989), followed by CS (8,729,729) and JG (7,747,130) (Table 5). After filtering MAF5, CS identified the most INDELs (915,255) followed by JG (907,983) and DT (904,512). INDELs were further divided into either insertions or deletions. Before filtering, JG identified the least insertions (2,625,087) and the second most deletions (5,122,043), and DT identified the most insertions (4,584,859) and deletions (7,554,130), while CS identified the second most insertions (3,788,665) and the least deletions (4,941,064). However, once the filtering was applied, JG identified the most insertions (412,220) and the least deletions (494,718), while CS had the second most of both insertions (399,858) and deletions (515,397), and DT reference identified the most deletions (522,313) and the least number of insertions (382,208).

### 3.7 Variant effects

The variants called against each reference likewise varied in the predicted impact effects. Of the three references, DT yielded the highest number of “high” impact variants (203,740), followed by CS and then JG with 142,821 and 69,846 variants, respectively (Table S8). This pattern followed for the other variant effect categories of “low,” “moderate,” and “modifier.” For the MAF5 filtered data, CS identified the largest number of predicted “high” impact variants (8591) followed by DT and JG with 3788 and 3205, respectively. This pattern likewise repeated for the “moderate” effect variants, with CS containing 60,463 variants, and DT and JG containing 37,052 and 35,431, respectively. However, for both “low” and “modifier,” DT yielded the most (48,472 and 18,815,074) followed by CS (38,309 and 15,316,243) and JG (27,194 and 14,427,274). Considering the variant effects within coding regions by functional class, missense, nonsense, and silent mutations, clear differences were observed between the references. For the combined count of the total number of variant effects, DT identified the most (3,527,680) followed by CS (3,019,779) and JG (2,000,277) before filtering. After filtering MAF5, CS identified the most (94,979) followed by DT (62,855) and JG (57,582). Based on the relative percentage of these variant effects, CS had a higher percentage of missense effects (63.11%) in comparison to DT (56.88%) or JG (60.17%). Similar trends were observed for the highest proportion of nonsense effects from CS (2.27%), in comparison to JG (1.6%) and DT (1.13%). CS also identified the least number of silent mutations (34.62%) in comparison to JG (38.24%) and DT (41.99%). Breaking down the effects further by region, it was observed that most variants were in the intergenic regions. However, DT had much less intergenic variations, instead having significantly more upstream, downstream, and transcript variations in comparison to CS and DT. Comparing JG and CS, it was also observed that JG had relatively more variants in the intergenic region (86.34%), while CS had more variants in the upstream, downstream, and both the coding (exon) and noncoding (intron) regions. JG also identified the least number of variants in the transcripts or gene regions.

### 3.8 VCF stats

After variant calling, a significant difference between the percentage of missing and heterozygous alleles was observed among the three reference genomes (Table S3). DT and CS had the highest percentage of missing alleles 16.98% and 16.21%, respectively, while JG had only 12.43% missing calls (Figure S5). All references identified a similar percentage of heterozygous alleles, although DT was nominally higher (13.94%) followed by CS (12.944%) and JG (12.43%). JG had significantly more reference alleles (67.30%) compared to the other two references, followed by CS (58.83%) and DT (51.85%).

### 3.9 Diversity

**FIGURE 5:**
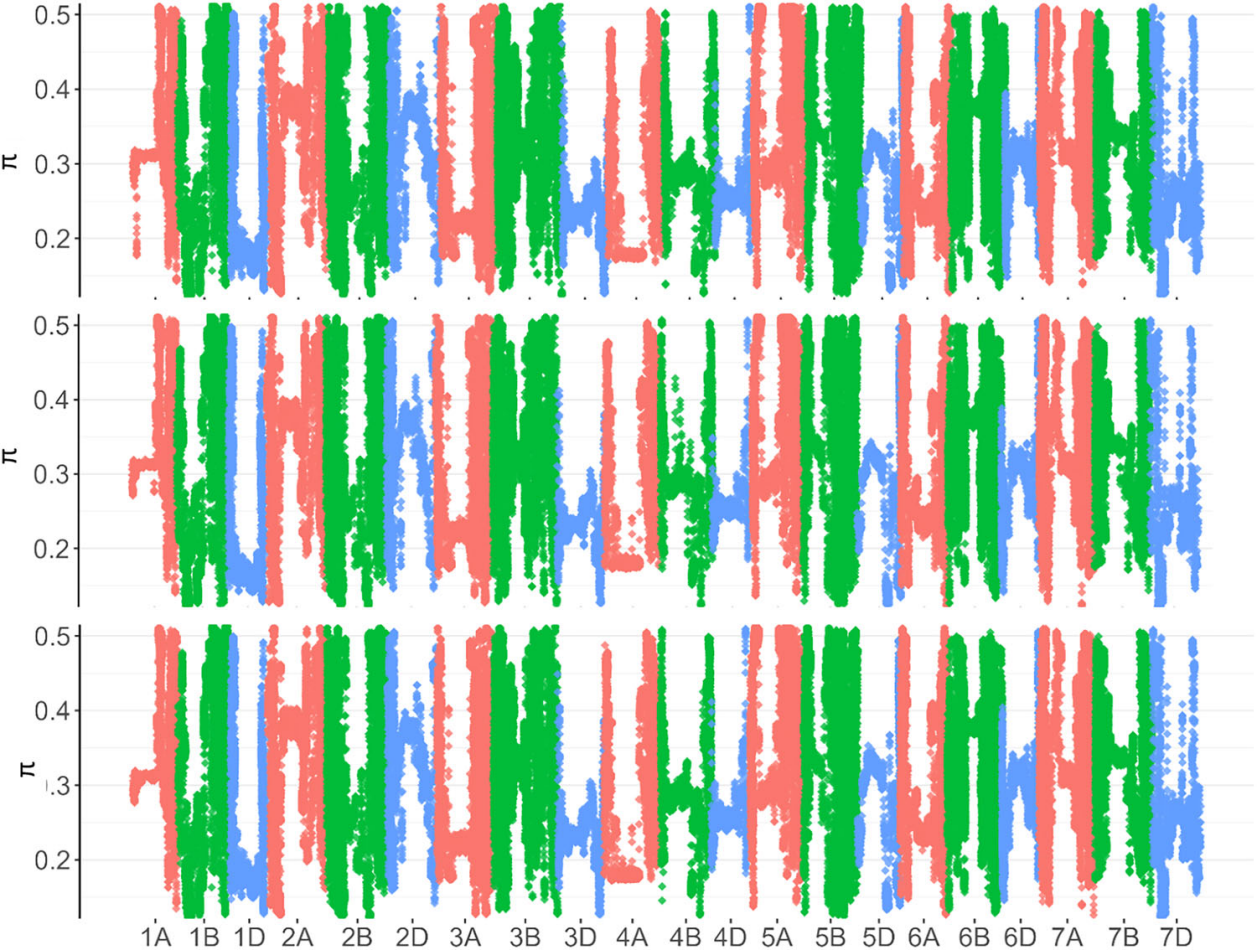
Allelic diversity as shown by metric Pi across the genome, with alignment to Jagger (top), Synthetic (middle), and Chinese Spring (bottom). The colors are representative sub‐genome, where red is genome A, green is genome B, and blue is genome D.

The phylogenetic tree and the allelic diversity (*π*) did not show any major differences among the three reference genomes (Figure 5). Based on the phylogenetic tree, genetic diversity of the panel and the relative similarities among the released varieties were visualized, which displayed the germplasm communication history of wheat breeding programs in the Great Plains (Figure S1). Genomic regions around the centromeres of each chromosome have much lower nucleotide diversity due to the historical less recombination events, while the two distal ends showed much higher Pi levels, reflecting previous research findings (The International Wheat Genome Sequencing Consortium, 2018; Choulet et al., 2014; Jordan et al., 2020). The only major discernible difference between the references was on the short arm of 1A in the JG reference, where there was a lower level of Pi trailing at the start of the Pi graph (Figure 5).

## 4 DISCUSSION

### 4.1 Reference synteny

**FIGURE 6:**
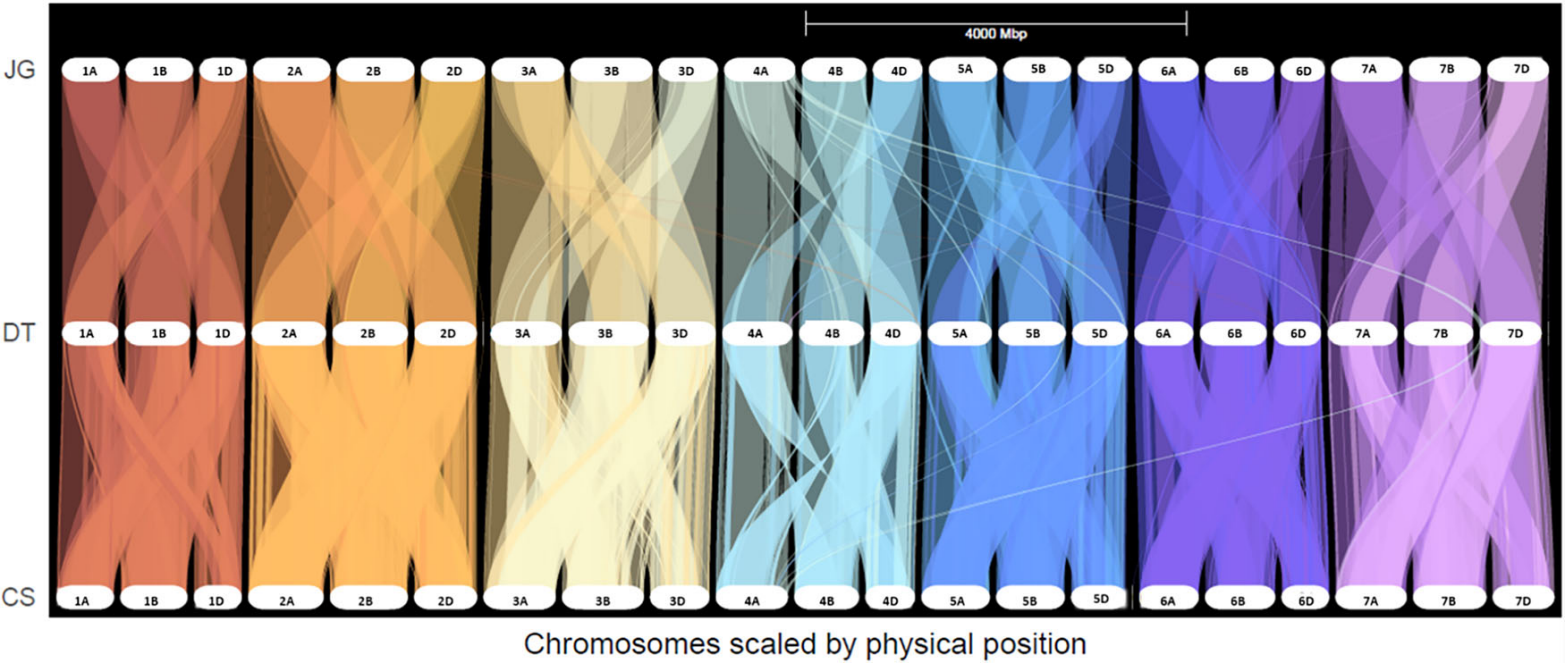
Whole genome synteny scaled by physical location. Colors across genomes represent the conserved synteny. Jagger (JG), Durum‐*tauschii* (DT), and Chinese Spring (CS) reference genomes.

The comparison of the three references, while not surprising, paints a picture of a smaller core genome with a large and diverse pan‐genome. Plant genomes are known to harbor many gene duplication events, and likewise, wheat is an allohexaploid crop, resulting from the combination of three different species (Dubcovsky & Dvorak, 2007). The evolutionary distance is proxied by the number of unique orthogroups. The DT reference represents a wild relative that continued its evolutionary path for many millennia after the hybridization event (*A. tauschii*), as well as a cultivated tetraploid wheat variety that has likewise been on its own domestication path from wild emmer wheat. The DT reference represents a combination of two different species with a large evolutionary gap from two hexaploid bread wheat cultivars CS and JG. Likewise, the distance between the CS and JG is evidence of the long history of plant breeding, and more specifically, plant breeding in the US Great Plains versus the 20th century of China. The core genome is represented by shared orthogroups present in all three references. In contrast, the pan‐genome would be orthogroups represented in either one or two other references. That is less than half of the orthogroups (48%) that are conserved across all three genomes, indicating the great potential of genetic diversity lost using a single reference. If a region of interest contains one of these orthogroups that are shared, researchers can look at the diversity in gene sequences to help identify the causal variant of the target trait. The evolution of modern wheat as we know it today is filled with translocations and other structural variations (Adhikari et al., 2022; Devos et al., 1995; Luo et al., 2007; Walkowiak et al., 2020; Zhou et al., 2021). Through looking at synteny mapping between references, large‐scale structural variants can be observed between the synthetic reference and the cultivated varieties (Figure 6).

The breaks of genetic colinearity reflect the areas that are highly divergent and unique to the specific reference (Figure S6). For example, the collinearity between JG and CS on chromosome 4A is very telling of the evolutionary trends over time. The dot plot and dual synteny plots (Figure S7) clearly show transversions between the two references, as well as a duplication of a region only present in JG. Granted, this is assuming that the assembled contigs of the references are accurate and not without problems. However, if only one reference can be deployed, and that reference does not contain the gene of interest as part of its genome, it may not be possible to identify the causal variation.

### 4.2 Reference genomes

The CS reference has been the gold standard for hexaploid wheat since its first draft release in 2014. However, CS is not a variety that closely resembles the cultivated varieties in the North American Great Plains region. Moreover, CS is a spring‐type wheat that has not been cultivated since the 1900s (Montenegro et al., 2017). For this reason, the popular Kansas winter wheat cultivar JG (Sears et al., 1997) was selected for its reference grade assembly that was released as part of the Wheat 10+ Genomes Project (Walkowiak et al., 2020) to compare the effects of alignment of WGS reads to a reference that most resembles the germplasms being aligned. The genetic distance between CS and JG can be visualized in the phylogenetic tree (Figure 1) where CS is much farther away than the rest of the cohort, while JG is much more closely related to the other cultivars. The synthetic reference (DT) was used as a nonrepresentative, poorly related reference genome. The first major difference between these reference genomes was the varied length of each chromosome. Sometimes, these extreme differences in chromosomal length may cause potential issues when trying to compare similar physical locations based on different references. Wheat is also known to have many structural variants (Saxena et al., 2014) such as insertions/deletions, and the discovery of these variants will be equally affected due to each reference having large variations in chromosome lengths. JG is also known to have a translocation introgression from the wild relative *A. ventricosa* and carries the 2N^v^S chromosomal segment (Gao et al., 2021). This segment is approximately 33 Mb in length and is present on the short arm of chromosome 2A in JG but not in CS. The 2N^v^S segment is known to harbor race‐specific resistance gene clusters *Yr17‐Lr37‐Sr38* (Helguera et al., 2003) and some additional race‐nonspecific genes (Rauf, Bajgain, et al., 2022; Rauf, Lan, et al., 2022), resistant to cereal cyst nematodes *Cre5* (Jahier et al., 2001) and even wheat blast *Magnaporthe oryzae* (*Triticum* pathotype) (Cruz et al., 2016). This resistance package was widely deployed until a virulence to *Yr17* was identified in 2008; however, a large portion of elite germplasm still has this segment in the Southern Great Plains region. JG has also been used as a core parent in many of the Great Plains breeding programs. This would make JG an ideal reference for lines, which are known to harbor this 2N^v^s segment, since possible variations would be lost if aligned to CS.

### 4.3 Alignments

The effects of the structural and genetic variation on NGS analysis begin to first present themselves looking at the alignment statistics. As expected, the DT reference tended to perform the poorest out of the three references. DT has statistically significantly more supplementary alignments (reads mapped to multiple locations) and paired reads on different chromosomes when compared to the other two references, revealing that DT least reflected the genomic characteristics of the germplasms aligned. Alternatively, JG had significantly lower reads with a mapping score of 0, base error rate, percentage of missing SNPs, and the highest percentage of reference SNPs. These metrics demonstrate that the JG reference most accurately represented the aligned germplasm, which is reflected in a lower base error rate, a higher percentage of reference alleles, and lower missing values. One interesting thing to highlight here is that there were no significant differences between references for the percentage of unmapped reads, number of duplicated reads, and average MQ. These metrics appeared more associated with the individual sample aligned, rather than the reference used. Since all three references are relatively similar, in that they represent hexaploid wheat rather than a different organism, the choice of reference does not have a significant impact on these metrics.

The aligned germplasm showed an affinity for the JG reference on the B sub‐genome where JG observed significantly higher chromosomal coverage overall. If a target trait of interest found a quantitative trait locus mapped to the B sub‐genome, then JG would be a strong choice to find the source of variation within this population. Although for the A genome, no reference was favored, and for the D genome, DT performed worse than the two cultivars, reflecting the differences in the cultivated hexaploid wheat D genome, and the wild *A. tauschii* accession. Even though important sources of genetic diversity have come from synthetic hexaploid introgressions, the cultivated D genome is still significantly different enough from the wild genome progenitor.

### 4.4 Variant discovery

Using either of these three references, researchers can observe the relative relationships between the cohort analyzed, resulting in almost identical phylogenetic trees and allelic diversity analysis. Binning variants by chromosomal segment helped to control the relative differences in physical positions and possible structural variation. For the raw variants, general trends across chromosomes seem to be conserved, except for example, the distal end of chromosome 2D, where the DT reference saw a substantial increase in variants compared to the other references (Figure S1). However, after filtering is applied, the relative differences between the references shrink quite significantly (Figure 4). For most of the chromosomal segments, the quantity of variants was more conserved, but we can observe some clear reference preferences, like in the significant drop in variants discovered in the distal end of 5A for DT when compared to the other references.

Using the raw variant files, we can capture the relative differences between the cohort evaluated and the reference itself. With DT being the most evolutionary distant from the cohort, DT identifies significantly more variants. The relative difference in the quantity of variants across the genome is dramatically reduced once filtering for minor allele frequency of 3%3 (MAF3) is applied, and it removes all monomorphic markers. Interestingly, when filtering for MAF3, DT still identifies more variants than JG. While all three references are relatively close to ∼30 million markers, DT identifies more markers at an MAF3. Since MAF3 allows for the identification of unique alleles in the population but removes the monomorphic markers, this would imply that DT can identify the most unique variants within the population. One hypothesis for the increase in variants for DT at MAF3 would be the repetitive use of synthetic hexaploids in the Great Plains germplasm. In this case, DT can identify rare alleles that are only present in a single sample, reflecting introgressions from synthetic hexaploids. However, since these alleles are rare in this cohort, they are removed when filtered at MAF5. MAF5 requires at least two individuals to have an alternative allele at a locus; thus, the most representative reference JG identifies the most variation between the evaluated cohort. Raw variants may be useful in understanding the genetic distance of the cohort to the reference, but they are less useful in understanding variation within this cohort. The initial discovery of more variants does not indicate more power in finding causal variation, but rather is bias imposed using a nonrepresentative reference. Under MAF5, JG allows for the highest level of variant discovery among the cohort being evaluated, while some of the resolution is lost using CS or DT.

### 4.5 Variant effects

When referencing the respective genome annotations, variants are disproportionately discovered in different features and SO classifications. This is assuming that the published annotation files are correct and not without errors. Depending on the research goals, the references showed a bias to the genetic locations and the predicted effects of that variant change in regard to the selected reference. JG had the highest proportion of intergenic variants (86.1%), while CS and DT observed higher exon, gene, intron, downstream, upstream, and transcript variants comparatively, with DT identifying the most variants in these locations in comparison to either JG or CS alone. DT likewise identified a large proportion of transcript variants (7.96%) in comparison to CS (<1% or 4278 variants) or JG (<1% or 474 variants) (Table S8). This would be attributed to the synthetic reference being a composite of two different species, rather than two distantly related organisms of the same species as JG and CS. This is likewise reflected in the number of variants classified into the gene regions, and for CS and JG, there are 17 and 4, respectively. However, for the DT reference, we observe 772 variants within gene regions, reflecting the genetic differences between two bread wheat cultivars and the synthetic reference. JG consistently had a lower number of high/low/moderate SO variant effects in comparison to the other references. This demonstrated that JG most accurately represents the germplasm aligned, as opposed to CS, which has a much higher rate of “high” effect variants. This revealed that the germplasm used in this study is highly divergent from CS in gene structure, likely due to its long genetic distance from the CS over years of artificial selection during breeding and genetic improvement in the North American Great Plains. Since CS is a landrace variety, the aligned germplasm reflects many more deleterious impacts when compared to CS. Since JG is a representative cultivar, it likewise harbors a similar evolutionary history, revealing fewer “high” impacts, since the aligned germplasm shares a similar breeding history. Although one would expect the DT reference to have the most number of predicted “high” effect variants, we observed CS with the highest for the filtered MAF5 set, while DT was just a little more than JG. One hypothesis that may explain this could be the continuous use of synthetic hexaploid wheat in germplasm improvement over the past several decades. This allowed multiple introgressions of cultivated *T. durum* and *A. tauschii* genetic materials into the common wheat breeding programs of the Southern Great Plains, resulting in increased similarity to DT over CS. These variant effects reflected the divergence of the elite germplasm and the earlier progenitors.

### 4.6 Discovery of introgressions

The most obvious single‐reference‐induced bias is the inability to visualize genomic information from large introgressions. The JG reference can be utilized to identify germplasm lines with the 2N^v^S segment on chromosome 2A. Gao et al. (2021) previously used the JG reference to screen historical germplasm lines. Using coverage data, we can confirm the presence or absence of the chromosomal segment 2N^v^S (Table S5). Lines with increased coverage on chromosome 2A by ∼2% using JG instead of CS indicate the presence of 2N^v^S (Figure S8). While lines without 2N^v^S had decreased coverage when aligned to JG rather than CS. These methods are similar to Keilwagen et al. (2022) who used coverage to identify other wild relative introgressions in modern wheat varieties. Otherwise, the development of diagnostic markers must be developed and deployed to understand simple PAV for any segment not in CS. While the introgression may be able to be linked to a variant that is discovered using an alternative reference, the actual variation in the region cannot be captured or analyzed. Researchers focusing on a trait within this introgression can only understand and study the variation within this segment if it is included in the reference genome when aligned.

Likewise, the identification of the 1RS rye chromosome translocation was identifiable by looking at the genome‐wide chromosome coverage statistics. For chromosomes that harbored the translocation, coverage was reduced by ∼23% when compared to the average coverage. Lines without the introgression showed some low‐quality alignments to a small portion of the rye chromosome, likely due to the presence of highly conserved sequences. However, the read depth and average quality of these alignments were below the minimum threshold of MQ > 20, so downstream processing would remove these reads. Lines with the translocation had an average MQ > 30, providing strong evidence of the rye chromosomal segment in the samples. While the presence of the translocation in many of these lines was known, two lines without publicly available marker data were demonstrated to harbor this translocation. This loss of a fifth of a chromosome is visible regardless of the reference used, but by not including the rye chromosome in an aligned reference, researchers are missing out on valuable sequence information.

The 1RS and 2N^v^S are relatively large introgressions ∼200 Mb and ∼33Mb, respectively. The 1RS was a significant portion of the chromosome, which can be identified as a large gap from our current references, while 2N^v^S is present inside of JG, allowing for identification by alignment to that portion of the genome. However, what if you had a known source of resistance in your germplasm? Could short reads from a target region be used to overcome the single‐reference bias against the discovery of resistance? *Gb3* came from a synthetic hexaploid with novel *A. tauschii* introgressions (Largo) (Azhaguvel et al., 2012; Joppa & Williams, 1982; Rudd et al., 2014). These resistance loci are absent from all three references, including the D genome donor to the DT reference (Azhaguvel et al., 2012).

The assembled tamGB3 contig was much smaller, around 2 Mb. While assembling contigs using only short reads is not very complete and inherently contains lots of gaps between sequence coverage, this tool can be useful to identify lines that contain a known beneficial haplotype. While this cohort only used three TAM lines, which are known to contain the *Gb3* gene and further evaluations will be required to ensure that the alignment coverage is due to *Gb3* and not compounded by population structure. The coverage analysis along tamGB3 allowed for the narrowing down of the possible window for where *Gb3* may be located. Long read sequencing would be required to ensure that *Gb3* was fully sequenced and present in the contig. However, this exercise demonstrates the value of high‐depth sequencing in the identification of genes that have yet to be cloned.

Not all programs have available WGS for all their research germplasm, but that may change as sequencing data costs decline and data become more readily available over time. This analysis can be a very useful tool in screening historic datasets or gene bank accessions for the presence or absence of known introgressions present and identifiable via reference genomes.

### 4.7 Reference genome effects

One of the biggest takeaways observed in this study is that single‐reference bias impacts variant discovery and downstream analysis. First, each reference has a specific structural variation and reference gene composition. There are limitations to what genes or orthogroups may be identified using aligned sequence information. With the core genome reflecting only 48% of the orthogroups, a large portion of the genetic variation is reference‐specific and may not be identified when aligning short reads to a reference without these unique genes. Initially, alignment revealed more variants depending on the dissimilarity of the reference to the cohort. However, this “higher resolution” of increased variants was removed when filtering out monomorphic or single‐sample alleles. After filtering, one is left with less variants to understand within cohort variation with the use of an unrepresentative reference. With an MAF3 filtering, we can observe any rare alleles with a synthetic hexaploid origin that are not present in either of the other references. The suitability of a reference to represent a cohort can likewise be evaluated by the percentage of reference alleles, heterozygous, and missing alleles of the raw variant files. Wheat is a highly homozygous self‐pollinated crop, and heterozygous calls tend to be errors in read alignment that could be the result of many circumstances such as hidden copy number variation or alignment of reads to homologous regions causing a heterozygous call. This is a similar problem that was previously identified in humans (Ballouz et al., 2019). With DT calling nominally more heterozygous calls, it demonstrates its lower suitability as a reference. Likewise, we observed that JG had lower missing calls and higher reference alleles than the other references, which demonstrated its higher suitability as a representative reference for the North American Great Plains wheat germplasm.

The true test of a reference is whether it can identify useful variation for researchers to answer their research questions. The discovery of SNPs and their relative locations on a reference genome is useful information. Often, when performing marker trait association analysis, researchers identify markers that are in linkage disequilibrium with the trait of interest. All the variation and genetic information that lies within these introgressions is filtered down to a few significant markers, which are linked to the presence of the introgression. If a reference containing the target trait is available, it is easy to achieve the genetic resolution to identify the true causal gene and understand the trait itself rather than just the location of an associated marker. While the 1R translocation is large enough to be identified due to the loss of a fifth of chromosome coverage, all the genetic information within that segment is lost. Variation occurring between lines, within this region, is likewise unable to be compared, since all the sequence information will be lost to unmapped reads. While markers may be found to be in strong linkage with the introgression, researchers are only seeing a signal linked to the variation as opposed to capturing the variation itself. 2N^v^S has been agronomically important for many years; however, researchers were limited to only identifying PAV. Many resistance traits were previously mapped to this region but have no visibility within the sequence. By using CS, researchers were limiting themselves to what is only visible with CS, but now JG can be useful to overcome this blind spot in research focused on this introgression.

The coverage analysis using the tamGB3 haplotype was a good test case to try to use short‐read whole genome sequence data to create a contig to screen other germplasms for traits of interest. Since this gene is not present in any of the three references, we needed to find a way to include it to overcome the single‐reference bias against the gene. Without long‐read sequencing data, one cannot be certain that *Gb3* is sequenced and contained inside the contig, but this can be a useful tool to help narrow down the window for where these genes of interest may be located. Further testing will be needed to test its application on a population with lower genotyping depth.

## 5 CONCLUSION

This study has demonstrated that the choice of reference can have a significant impact on variant discovery. The utilization of the most representative reference genome could facilitate the accurate determination of causal variants and provide a better understanding of the genetic architecture of the experimental population. It is important to understand where candidate reference genomes are highly conserved or divergent to inform the choice when selecting the best reference for specific research questions. Likewise, a reference genome containing an introgression or haplotype of interest will allow for more accurate identification in an aligned population, as well as aid in the identification of the underlying genetic source of the phenotype.

Reference bias may be able to be overcome in the pan‐genomics era. Tools such as Vg‐ Giraffe (Sirén et al., 2021), allowing for the construction of nonlinear genomic graphs which can incorporate multiple haplotypes to form a pan‐genome, become a more popular solution to a single reference may be on the horizon. Understanding the bias from using a single reference can help researchers to make the best choice for their research goals. Ultimately, the use of pan‐genomics will become routine in future variant discovery and help to leverage more genetic backgrounds to overcome the single‐reference bias.

## AUTHOR CONTRIBUTIONS

**Kyle Parker**: Conceptualization; data curation; formal analysis; investigation; methodology; software; validation; visualization; writing—original draft; writing—review and editing. **Zhen Wang**: Software; validation; visualization; writing—review and editing. **Yahya Rauf**: Validation; visualization; writing—review and editing. **Liangliang Gao**: Data curation; resources; software; validation; writing—review and editing. **Jesse Poland**: Resources; software; validation; visualization; writing—review and editing. **Jackie Rudd**: Funding acquisition; resources; writing—review and editing. **Amir Ibrahim**: Funding acquisition; resources; supervision; writing—review and editing. **Shuyu Liu**: Conceptualization; funding acquisition; investigation; project administration; resources; supervision; writing—original draft; writing—review and editing.

## CONFLICT OF INTEREST STATEMENT

The authors declare no conflicts of interest.

## Supporting information



Supplementary Material

Supplementary Material

## Data Availability

Sequence data for the 29 Great Plains Germplasm lines are available as part of the NCBI BioProject PRJNA694980. Chinese Spring short read sequencing data are in the NCBI BioProject PRJNA393343. Jagger short read sequencing data are in the NCBI BioProject PRJNA544491. The tamGB3 contig, DT fasta reference, and DT raw VCF file will be available at https://doi.org/10.5281/zenodo.10801851. The CS and JG raw VCF files will be available at https://doi.org/10.5281/zenodo.10801971. Variant calling workflow NexGenSnake (https://github.com/Kxpark/NexGenSnake). Other datasets used and/or analyzed during the current study are available from the corresponding author on reasonable request.
