# Supplementary material for: Diverse reference genomes detect variants in the US winter wheat
Source: Plant Genome. 2025 Dec 30;19(1):e70160. doi: 10.1002/tpg2.70160 (PMC12750494; doi:10.1002/tpg2.70160)
Supplement: Supplementary file 2 [file TPG2-19-e70160-s002.docx]

## **Supplementary tables**

| Table S1. List of 31 Wheat Cultivars and lines of Great Plains Hard Winter Wheat | | | | | |
| --- | --- | --- | --- | --- | --- |
| Cultivar | SRR number | Size of Fastq | Species | Origin | Fold^1^ |
| Jagger | SRR8255599 | 102.7 G | Triticum aestivum | Kansas | 7x |
| Chinese Spring | SRR5893652 | 132.45 | Triticum aestivum | China | 10x |
| SY Monument | SRR13572411 | 111.40 G | Triticum aestivum | Syngenta | 8X |
| Duster | SRR13572412 | 148.48 G | Triticum aestivum | Oklahoma | 11X |
| TAM112 | SRR13572413 | 308.70 G | Triticum aestivum | Texas | 22X |
| TAM107 | SRR13572414 | 122.28 G | Triticum aestivum | Texas | 9X |
| Karl92 | SRR13572415 | 145.55 G | Triticum aestivum | Kansas | 10X |
| KS96WGRC40 | SRR13572416 | 70.84 G | Triticum aestivum | Kansas | 5X |
| WBCedar | SRR13572419 | 138.46 G | Triticum aestivum | WestBread | 10X |
| KS030923K-5 | SRR13572422 | 141.26 G | Triticum aestivum | Kansas | 10X |
| Larry | SRR13572423 | 335.51 G | Triticum aestivum | Kansas | 24X |
| Overley | SRR13572424 | 166.27 G | Triticum aestivum | Kansas | 12X |
| ClaraCL | SRR13572425 | 87.94 G | Triticum aestivum | Kansas | 6X |
| KS060476M-6 | SRR13572426 | 118.46 G | Triticum aestivum | Kansas | 8X |
| TAM111 | SRR13572427 | 85.02 G | Triticum aestivum | Texas | 6X |
| TAM204 | SRR13572428 | 196.18 G | Triticum aestivum | Texas | 14X |
| Heyne | SRR13572429 | 97.10 G | Triticum aestivum | Kansas | 7X |
| KanMark | SRR13572430 | 103.44 G | Triticum aestivum | Kansas | 7X |
| HotRod | SRR13572432 | 81.40 G | Triticum aestivum | Kansas | 6X |
| TAM114 | SRR13572433 | 100.25 G | Triticum aestivum | Texas | 7X |
| TAM205 | SRR13572434 | 181.49 G | Triticum aestivum | Texas | 13X |
| 2145 | SRR13572435 | 184.03 G | Triticum aestivum | Kansas | 13X |
| Gallagher | SRR13572436 | 124.57 G | Triticum aestivum | Oklahoma | 9X |
| WB Redhawk | SRR13572437 | 129.47 G | Triticum aestivum | WestBread | 9X |
| Aspen | SRR13572438 | 131.42 G | Triticum aestivum | WestBread | 9X |
| Ruby-Lee | SRR13572439 | 146.50 G | Triticum aestivum | Oklahoma | 10X |
| 1863 | SRR13572440 | 122.20 G | Triticum aestivum | Kansas | 9X |
| TAM115 | SRR13572441 | 180.34 G | Triticum aestivum | Texas | 13X |
| Everest | SRR13572443 | 111.19 G | Triticum aestivum | Kansas | 8X |
| KS061470M-4 | SRR13572444 | 84.26 G | Triticum aestivum | Kansas | 6X |
| Jagalene | SRR13572445 | 94.36 G | Triticum aestivum | Kansas | 7X |
| ^1^Fold coverage was calculated by dividing the raw sequence files by 14G for the length of the hexaploid wheat genome. | | | | | |

| Table S2. Variants Discovered by Reference Genome. | | | | | | | | | | | | |  |
| --- | --- | --- | --- | --- | --- | --- | --- | --- | --- | --- | --- | --- | --- |
|  | References | | | Unfiltered | | | Filtered MAF 3% | | | Filtered MAF 5% | | |  |
|  | JG | CS | DT | JG | CS | DT | JG | CS | DT | JG | CS | DT | |
| Chromosome | Length | Length | Length | Variants | Variants | Variants | Variants | Variants | Variants | Variants | Variants | Variants | |
| 1A | 596,211,899 | 598,660,471 | 585,266,722 | 7,751,558 | 9,947,091 | 10,622,265 | 1,113,837 | 1,082,883 | 1,132,376 | 785,401 | 749,458 | 749,240 | |
| 1B | 705,338,699 | 700,547,350 | 681,112,512 | 11,603,069 | 13,370,611 | 16,228,488 | 1,767,647 | 1,717,533 | 1,722,999 | 932,755 | 930,805 | 849,512 | |
| 1D | 493,450,010 | 498,638,509 | 501,967,303 | 8,688,778 | 8,981,160 | 13,402,433 | 1,143,184 | 1,120,128 | 1,160,479 | 310,414 | 303,560 | 284,291 | |
| 2A | 804,285,258 | 787,782,082 | 775,448,786 | 11,541,817 | 13,049,270 | 13,788,043 | 1,926,122 | 1,806,241 | 1,896,946 | 1,084,843 | 1,013,834 | 980,262 | |
| 2B | 810,734,643 | 812,755,788 | 790,338,525 | 13,155,806 | 15,657,540 | 17,628,054 | 2,430,324 | 2,346,874 | 2,366,949 | 832,805 | 820,073 | 806,943 | |
| 2D | 673,981,989 | 656,544,405 | 650,458,083 | 8,246,842 | 8,728,503 | 13,098,208 | 968,242 | 999,466 | 1,114,595 | 209,098 | 223,003 | 198,734 | |
| 3A | 754,496,630 | 754,128,162 | 746,673,839 | 9,034,244 | 10,551,544 | 11,824,054 | 1,745,034 | 1,667,048 | 1,732,367 | 883,669 | 831,288 | 834,125 | |
| 3B | 855,759,449 | 851,934,019 | 836,514,780 | 15,579,882 | 17,506,025 | 19,456,602 | 2,552,339 | 2,482,805 | 2,580,980 | 980,449 | 962,363 | 910,215 | |
| 3D | 614,042,580 | 619,618,552 | 627,456,150 | 6,943,933 | 7,601,601 | 12,376,409 | 971,900 | 946,328 | 1,028,905 | 217,388 | 210,697 | 177,791 | |
| 4A | 743,847,818 | 754,227,511 | 736,872,137 | 9,911,031 | 10,954,355 | 12,128,590 | 1,843,323 | 1,929,388 | 1,996,827 | 1,217,601 | 1,168,170 | 1,147,443 | |
| 4B | 673,340,788 | 673,810,255 | 676,292,951 | 7,784,871 | 8,834,253 | 13,571,389 | 1,349,613 | 1,348,523 | 1,333,365 | 472,479 | 459,590 | 399,527 | |
| 4D | 515,668,560 | 518,332,611 | 525,206,139 | 5,407,736 | 5,767,886 | 9,134,526 | 654,698 | 631,287 | 773,964 | 71,992 | 70,200 | 61,512 | |
| 5A | 713,320,806 | 713,360,525 | 669,155,517 | 8,081,742 | 9,613,184 | 10,448,530 | 1,192,628 | 1,162,141 | 1,149,098 | 546,988 | 532,485 | 473,909 | |
| 5B | 703,598,484 | 714,805,278 | 701,372,996 | 11,353,739 | 13,360,791 | 14,648,508 | 1,865,761 | 1,868,264 | 1,960,211 | 656,280 | 655,501 | 610,453 | |
| 5D | 570,159,854 | 569,951,140 | 576,238,907 | 5,793,496 | 6,255,889 | 11,156,105 | 811,555 | 784,116 | 855,076 | 125,275 | 122,475 | 106,962 | |
| 6A | 625,793,224 | 622,669,697 | 615,672,275 | 7,829,740 | 8,791,741 | 9,745,830 | 1,284,306 | 1,331,617 | 1,373,466 | 735,765 | 769,331 | 743,227 | |
| 6B | 721,110,502 | 731,188,232 | 698,614,761 | 12,055,173 | 14,453,641 | 15,167,921 | 2,067,146 | 1,967,310 | 2,051,974 | 963,852 | 936,721 | 905,998 | |
| 6D | 459,355,444 | 495,380,293 | 495,363,004 | 4,542,378 | 4,846,356 | 9,069,779 | 569,807 | 562,948 | 682,011 | 133,962 | 142,209 | 130,425 | |
| 7A | 745,201,001 | 744,491,536 | 728,031,845 | 10,005,106 | 11,723,693 | 13,655,315 | 1,597,437 | 1,563,295 | 1,593,748 | 705,776 | 675,876 | 657,941 | |
| 7B | 749,284,433 | 764,081,788 | 722,970,987 | 10,593,269 | 12,462,154 | 15,722,763 | 1,597,765 | 1,559,230 | 1,596,100 | 534,502 | 513,098 | 480,177 | |
| 7D | 643,344,672 | 642,921,167 | 644,841,383 | 7,944,111 | 8,284,897 | 12,909,296 | 1,461,274 | 1,418,711 | 1,406,322 | 338,225 | 333,581 | 284,256 | |
| Total | 14,172,326,743 | 14,225,829,371 | 13,985,869,602 | 193,848,321 | 222,385,125 | 275,783,108 | 30,913,942 | 30,415,536 | 31,508,758 | 12,739,519 | 12,424,318 | 11,792,943 | |
| Chinese Spring (CS), Synthetic (DT), and Jagger (JG) reference genomes. The lengths of the chromosomes are in base pairs, and filtered datasets consisted of filtering for <10% missing, <10% heterozygosity, and minor allele frequency of 3% and 5%. | | | | | | | | | | | | |  |
|  | | | | | | | | | | | | |  |

| Table S3. Alignment Statistics and Variance Calls. | | | | | | | |  |
| --- | --- | --- | --- | --- | --- | --- | --- | --- |
| Metric | Referenceᶤ | Group¹ | Mean ± SD² | Metric | Reference | Group | Mean ± SD |  |
| Unmapped Reads | JG | A | 1.173 ± 0.468 | Mapping Quality | CS | A | 35.259 ± 1.044 |  |
|  | DT | A | 1.126 ± 0.329 |  | JG | A | 35.248 ± 1.062 |  |
|  | CS | A | 1.074 ± 0.456 |  | DT | A | 35.162 ± 0.955 |  |
| Supplementary Aligned Reads | DT | A | 0.871 ± 0.153 | Reads Paired on Different Chromosome | DT | A | 1.163 ± 0.819 |  |
|  | JG | B | 0.61 ± 0.122 |  | CS | B | 0.903 ± 0.577 |  |
|  | CS | B | 0.597 ± 0.109 |  | JG | B | 0.896 ± 0.64 |  |
| Duplicated Reads | JG | A | 6.06 ± 3.441 | Overall Coverage | JG | A | 87.6 ± 4.581 |  |
|  | DT | A | 6.039 ± 3.513 |  | CS | A | 87.2 ± 4.564 |  |
|  | CS | A | 5.402 ± 3.51 |  | DT | B | 85.243 ± 3.934 |  |
| Properly Paired Reads | CS | A | 96.166 ± 2.344 | Heterozygous Calls | DT | A | 13.936 ± 2.59 |  |
|  | JG | AB | 95.941 ± 2.494 |  | CS | A | 12.943 ± 2.529 |  |
|  | DT | B | 95.455 ± 2.836 |  | JG | A | 12.427 ± 3.221 |  |
| Reads with Mapping Quality of 0 | DT | A | 16.38 ± 2.169 | Missing Calls | DT | A | 16.979 ± 3.863 |  |
|  | CS | A | 15.843 ± 2.354 |  | CS | A | 16.209 ± 3.948 |  |
|  | JG | B | 12.915 ± 2.356 |  | JG | B | 12.188 ± 3.546 |  |
| Base error rate | DT | A | 0.013 ± 0.001 | Reference Calls | JG | A | 67.3 ± 4.657 |  |
|  | CS | B | 0.01 ± 0.001 |  | CS | B | 58.826 ± 2.943 |  |
|  | JG | C | 0.009 ± 0.001 |  | DT | C | 51.849 ± 2.839 |  |
| ᶤReference is the respective reference genome used. JG is Jagger, DT is Synthetic, and CS is Chinese Spring. Group, references with the same letter are not significantly different to a 95% confidence level. ²Mean ± SD is the average percentage of reads, plus or minus the standard deviation. | | | | | | | |  |
|  |  |  |  |  |  |  |  |  |

| Table S4. Chromosome Coverage by Reference. | | | | | | | |  |
| --- | --- | --- | --- | --- | --- | --- | --- | --- |
| Regionᶳ | Referenceᶤ | Group¹ | Mean ± SD² | Region | Reference | Group | Mean ± SD |  |
| 1A | JG | A | 84.284 ± 10.268 | 5A | JG | A | 88.764 ± 3.102 |  |
|  | DT | A | 83.12 ± 8.787 |  | CS | A | 87.887 ± 3.169 |  |
|  | CS | A | 82.655 ± 9.637 |  | DT | A | 87.577 ± 2.866 |  |
| 1B | JG | A | 85.593 ± 5.58 | 5B | DT | A | 85.74 ± 2.914 |  |
|  | CS | B | 83.331 ± 5.163 |  | CS | A | 85.668 ± 3.145 |  |
|  | DT | B | 81.797 ± 4.678 |  | JG | A | 84.738 ± 3.427 |  |
| 1D | CS | A | 88.523 ± 4.076 | 5D | JG | A | 90.247 ± 2.89 |  |
|  | JG | A | 88.178 ± 4.322 |  | CS | A | 90.002 ± 2.932 |  |
|  | DT | B | 82.574 ± 3.82 |  | DT | B | 84.222 ± 3.029 |  |
| 2A | DT | A | 86.832 ± 2.868 | 6A | CS | A | 87.132 ± 2.994 |  |
|  | CS | A | 86.567 ± 3.401 |  | DT | A | 87.112 ± 2.959 |  |
|  | JG | A | 85.705 ± 3.865 |  | JG | A | 85.69 ± 3.098 |  |
| 2B | JG | A | 85.682 ± 3.518 | 6B | JG | A | 87.167 ± 3.351 |  |
|  | CS | AB | 83.83 ± 3.224 |  | DT | A | 85.291 ± 2.81 |  |
|  | DT | B | 83.24 ± 3.291 |  | CS | B | 82.975 ± 3.131 |  |
| 2D | CS | A | 88.804 ± 3.069 | 6D | JG | A | 90.663 ± 2.793 |  |
|  | DT | B | 85.042 ± 2.841 |  | CS | A | 89.998 ± 2.908 |  |
|  | JG | B | 84.106 ± 3.602 |  | DT | B | 85.272 ± 2.968 |  |
| 3A | JG | A | 89.296 ± 3.045 | 7A | JG | A | 87.223 ± 3.095 |  |
|  | CS | A | 88.621 ± 2.932 |  | CS | AB | 86.452 ± 3.119 |  |
|  | DT | A | 87.915 ± 2.844 |  | DT | B | 85.262 ± 3.064 |  |
| 3B | JG | A | 86.041 ± 3.91 | 7B | JG | A | 88.061 ± 3.157 |  |
|  | DT | A | 85.189 ± 3.088 |  | CS | B | 85.896 ± 2.999 |  |
|  | CS | A | 84.376 ± 3.255 |  | DT | B | 84.497 ± 2.978 |  |
| 3D | JG | A | 90.157 ± 3.483 | 7D | CS | A | 90.047 ± 2.834 |  |
|  | CS | A | 89.403 ± 3.375 |  | JG | A | 89.753 ± 2.867 |  |
|  | DT | B | 84.595 ± 3.158 |  | DT | B | 83.479 ± 3.029 |  |
| 4A | CS | A | 87.211 ± 3.087 | **A** | JG | A | 86.756 ± 5.119 |  |
|  | DT | A | 86.83 ± 2.968 |  | CS | A | 86.646 ± 4.912 |  |
|  | JG | A | 86.329 ± 3.266 |  | DT | A | 86.378 ± 4.495 |  |
| 4B | CS | A | 90.608 ± 2.714 | **B** | JG | A | 86.825 ± 4.137 |  |
|  | JG | A | 90.491 ± 2.833 |  | CS | B | 85.241 ± 4.181 |  |
|  | DT | B | 87.153 ± 2.85 |  | DT | B | 84.701 ± 3.619 |  |
| 4D | JG | A | 91.44 ± 2.946 | **D** | CS | A | 89.713 ± 3.25 |  |
|  | CS | A | 91.212 ± 2.945 |  | JG | A | 89.22 ± 3.986 |  |
|  | DT | B | 87.373 ± 2.804 |  | DT | B | 84.651 ± 3.369 |  |
| ᶳregion is the segment of genome analyzed, where the single **A, B, D** letter designation is the sub-genome overall. ᶤReference is the respective reference genome used. JG is Jagger, DT is Synthetic, and CS is Chinese Spring. ¹Group, references with the same letter are not significantly different to a 95% confidence level. ²Mean ± SD is the overall mean chromosome coverage in percent coverage, plus or minus the standard deviation. | | | | | | | |  |
|  |  |  |  |  |  |  |  |  |

| Table S5. Alignment Coverage of 2NvS. | | | | | | | | |
| --- | --- | --- | --- | --- | --- | --- | --- | --- |
|  | **Line** | **Refᶳ** | **Chromᶤ** | **Reads** | **Cov¹ bases** | **Coverage** | **∆ Cov** | **2NvS²** |
|  | **1863** | CS | 2A | 31333679 | 682874373 | 86.6832 | -3.7776 | Yes |
|  |  | JG | 2A | 31812712 | 727562748 | 90.4608 |  |  |
|  | **Duster** | CS | 2A | 37261786 | 692112401 | 87.8558 | 6.8938 | No |
|  |  | JG | 2A | 23526887 | 651165107 | 80.962 |  |  |
|  | **Gallagher** | CS | 2A | 33155087 | 696106422 | 88.3628 | 1.636 | No |
|  |  | JG | 2A | 31116862 | 697530557 | 86.7268 |  |  |
|  | **Heyne** | CS | 2A | 24844885 | 653614293 | 82.9689 | -1.9515 | Yes |
|  |  | JG | 2A | 24801241 | 683002328 | 84.9204 |  |  |
|  | **Jagalene** | CS | 2A | 24410639 | 660966344 | 83.9022 | -2.7763 | Yes |
|  |  | JG | 2A | 24222306 | 697142522 | 86.6785 |  |  |
|  | **KS030923K-5** | CS | 2A | 35140892 | 680522342 | 86.3846 | -1.4036 | Predicted |
|  |  | JG | 2A | 34926199 | 706067891 | 87.7882 |  |  |
|  | **KS060476M-6** | CS | 2A | 29295642 | 662825916 | 84.1382 | -2.0742 | Yes |
|  |  | JG | 2A | 29359105 | 693393660 | 86.2124 |  |  |
|  | **KS061470M-4** | CS | 2A | 20375129 | 622144551 | 78.9742 | -2.0495 | Yes |
|  |  | JG | 2A | 20636707 | 651661788 | 81.0237 |  |  |
|  | **Larry** | CS | 2A | 84190022 | 723893136 | 91.89 | -1.8401 | Yes |
|  |  | JG | 2A | 82598923 | 753857344 | 93.7301 |  |  |
|  | **Overley** | CS | 2A | 41569202 | 696221738 | 88.3775 | -2.8739 | Yes |
|  |  | JG | 2A | 41592260 | 733921210 | 91.2514 |  |  |
|  | **SY Monument** | CS | 2A | 27540505 | 659993519 | 83.7787 | -2.3051 | Predicted |
|  |  | JG | 2A | 27771319 | 692359633 | 86.0838 |  |  |
|  | **TAM 107** | CS | 2A | 31043779 | 678866525 | 86.1744 | 3.1441 | No |
|  |  | JG | 2A | 28758376 | 667800515 | 83.0303 |  |  |
|  | **TAM 111** | CS | 2A | 22360443 | 660571695 | 83.8521 | 2.4815 | No |
|  |  | JG | 2A | 21507024 | 654451690 | 81.3706 |  |  |
|  | **TAM 112** | CS | 2A | 72750281 | 728734093 | 92.5045 | 3.192 | No |
|  |  | JG | 2A | 67609704 | 718327078 | 89.3125 |  |  |
|  | **TAM 114** | CS | 2A | 25409737 | 663023997 | 84.1634 | 2.161 | No |
|  |  | JG | 2A | 24463173 | 659533294 | 82.0024 |  |  |
|  | **TAM 115** | CS | 2A | 46358158 | 707231455 | 89.775 | 2.2784 | No |
|  |  | JG | 2A | 44234163 | 703722138 | 87.4966 |  |  |
|  | **TAM 204** | CS | 2A | 47954507 | 690275237 | 87.6226 | -2.6149 | Yes |
|  |  | JG | 2A | 49198230 | 725766530 | 90.2375 |  |  |
|  | **TAM 205** | CS | 2A | 43242948 | 705498672 | 89.5551 | 2.9153 | No |
|  |  | JG | 2A | 40631338 | 696830857 | 86.6398 |  |  |
|  | Reads are individual reads aligned to the chromosome. Coverage is calculated by the percentage of covered bases on chromosome 2A. ᶳRef being Reference genome of Chinese Spring (CS) and Jagger (JG). Reads are individual reads aligned to the chromosome. ¹Cov is short for coverage which calculated by the percentage of covered bases on chromosome 2A. ∆ means change. ²Yes and no values are confirmed by marker screening data, while predicted are unconfirmed but predicted based on coverage. | | | | | | | |

| Table S6. Alignment Coverage of Rye Translocations. | | | | | | | | | | | | | | |
| --- | --- | --- | --- | --- | --- | --- | --- | --- | --- | --- | --- | --- | --- | --- |
| **Line** | **Refᶤ** | **Chromᶳ** | **Reads** | **Covered bases** | **Cov¹** | **Read Depth** | **Chrom MQ²** | **Average Cov** | **Average Read Depth** | **Average MQ** | **Δ Cov** | **Δ Read Depth** | **Δ MQ** | **1R^§^** |
| **Aspen** | JG:1R | 1A | 18,177,336 | 383,023,879 | 64.2 | 4.6 | 38.2 | 88.0 | 6.4 | 43.6 | -23.7 | -1.8 | -5.4 | 1A:1RS |
|  |  | 1R | 13,505,107 | 295,576,650 | 31.4 | 2.0 | 30.3 |  |  |  |  |  | -13.3 |  |
| **Duster** | JG:1R | 1A | 28,879,993 | 537,447,200 | 90.1 | 7.3 | 42.7 | 89.0 | 7.2 | 43.7 | 1.1 | 0.1 | -1.0 | No |
|  |  | 1R | 2,208,294 | 18,348,650 | 1.9 | 0.2 | 8.8 |  |  |  |  |  | -34.9 |  |
| **Gallagher** | JG:1R | 1B | 22,467,116 | 458,605,861 | 65.0 | 4.8 | 39.3 | 88.6 | 6.2 | 44.7 | -23.6 | -1.4 | -5.4 | 1B:1RS |
|  |  | 1R | 12,717,661 | 293,127,513 | 31.2 | 1.9 | 31.5 |  |  |  |  |  | -13.2 |  |
| **WBCedar** | JG:1R | 1A | 19,215,949 | 388,268,786 | 65.1 | 4.8 | 39.6 | 89.0 | 6.8 | 44.8 | -23.9 | -2.0 | -5.2 | predict |
|  |  | 1R | 14,178,443 | 302,865,301 | 32.2 | 2.2 | 31.3 |  |  |  |  |  | -13.5 |  |
| **Jagalene** | JG:1R | 1A | 18,215,963 | 524,295,801 | 87.9 | 4.6 | 45.1 | 86.4 | 4.6 | 45.7 | 1.5 | 0.0 | -0.6 | No |
|  |  | 1R | 1,316,598 | 13,038,873 | 1.4 | 0.1 | 10.6 |  |  |  |  |  | -35.1 |  |
| **Larry** | JG:1R | 1B | 57,373,985 | 507,346,272 | 71.9 | 12.0 | 42.8 | 93.5 | 15.4 | 47.4 | -21.6 | -3.4 | -4.6 | 1B:1RS |
|  |  | 1R | 32,044,743 | 390,839,934 | 41.5 | 4.9 | 32.3 |  |  |  |  |  | -15.1 |  |
| **TAM 107** | JG:1R | 1A | 16,917,846 | 383,029,067 | 64.2 | 4.2 | 38.1 | 87.8 | 6.0 | 43.8 | -23.6 | -1.8 | -5.7 | 1A:1RS |
|  |  | 1R | 12,318,346 | 288,867,571 | 30.7 | 1.9 | 30.4 |  |  |  |  |  | -13.4 |  |
| **TAM 112** | JG:1R | 1A | 37,804,985 | 424,155,617 | 71.1 | 9.3 | 41.4 | 92.6 | 13.6 | 45.6 | -21.5 | -4.3 | -4.2 | 1A:1RS |
|  |  | 1R | 27,613,105 | 386,033,868 | 41.0 | 4.2 | 31.5 |  |  |  |  |  | -14.1 |  |
| **TAM 114** | JG:1R | 1A | 19,899,943 | 518,502,004 | 87.0 | 5.0 | 43.4 | 86.0 | 5.0 | 44.4 | 1.0 | 0.0 | -1.0 | No |
|  |  | 1R | 1,316,719 | 13,008,237 | 1.4 | 0.2 | 9.3 |  |  |  |  |  | -35.1 |  |
| **TAM 115** | JG:1R | 1A | 24,918,005 | 400,451,120 | 67.2 | 6.2 | 39.6 | 90.3 | 8.8 | 44.4 | -23.1 | -2.6 | -4.8 | 1A:1RS |
|  |  | 1R | 18,021,030 | 318,080,211 | 33.8 | 2.7 | 31.4 |  |  |  |  |  | -13.0 |  |
| **TAM 204** | JG:1R | 1A | 38,324,845 | 547,279,728 | 91.8 | 9.6 | 42.9 | 90.6 | 9.5 | 43.9 | 1.2 | 0.1 | -1.0 | No |
|  |  | 1R | 3,322,826 | 24,657,472 | 2.6 | 0.4 | 10.7 |  |  |  |  |  | -33.2 |  |
| **KS96WGRC40** | JG:1R | 1A | 8,236,618 | 350,513,243 | 58.8 | 2.1 | 41.6 | 81.5 | 3.0 | 44.8 | -22.7 | -0.9 | -3.2 | Predict |
|  |  | 1R | 6,186,138 | 264,444,212 | 28.1 | 1.0 | 31.7 |  |  |  |  |  | -13.1 |  |
| ᶤRef is for Reference, and Jagger Rye (JG:1R) reference genome is the Jagger reference with rye chromosome 1 included. ᶳChrom is the respective chromosome being analyzed with 1R being the Rye chromosome 1, while 1A and 1B are hexaploid wheat chromosomes. ¹Cov as an abbreviation for chromosome coverage and Coverage is calculated by the percentage of covered bases on chromosome. MQ is mapping quality in terms of phred score. Reads are individual reads aligned to the chromosome. With Δ meaning the change. **^§^**Yes and no values are confirmed by marker screening data, while predicted are unconfirmed but predicted based on coverage. | | | | | | | | | | | | | | |

|  | Table S7. Alignment Coverage of tamGB3. | | | | | | |
| --- | --- | --- | --- | --- | --- | --- | --- |
|  | **Line** | **Chromᶳ** | **Reads** | **Covᶤ bases** | **Coverage** | **Δ Cov** | **GB3¹** |
|  | **Aspen** | 7D | 40301926 | 611845154 | 95.2 | 30.0 | No |
|  |  | tamGB3 | 56640 | 1870998 | 65.2 |  |  |
|  | **Duster** | 7D | 45963717 | 614880764 | 95.6 | 27.9 | No |
|  |  | tamGB3 | 63992 | 1943811 | 67.7 |  |  |
|  | **Gallagher** | 7D | 37906802 | 610236551 | 94.9 | 30.9 | No |
|  |  | tamGB3 | 51984 | 1836388 | 64.0 |  |  |
|  | **WBCedar** | 7D | 42009621 | 613740718 | 95.5 | 29.7 | No |
|  |  | tamGB3 | 59878 | 1886952 | 65.7 |  |  |
|  | **Jagalene** | 7D | 28948370 | 594477733 | 92.5 | 32.5 | No |
|  |  | tamGB3 | 41251 | 1720153 | 59.9 |  |  |
|  | **Larry** | 7D | 99425594 | 622661669 | 96.8 | 24.1 | No |
|  |  | tamGB3 | 143270 | 2087622 | 72.7 |  |  |
|  | **TAM 107** | 7D | 37804716 | 606958680 | 94.4 | 30.2 | No |
|  |  | tamGB3 | 52343 | 1841846 | 64.2 |  |  |
|  | **TAM 112** | 7D | 88244148 | 616047250 | 95.8 | 8.1 | Yes |
|  |  | tamGB3 | 214920 | 2518460 | 87.7 |  |  |
|  | **TAM 114** | 7D | 30798761 | 596140024 | 92.7 | 31.8 | No |
|  |  | tamGB3 | 42044 | 1749613 | 60.9 |  |  |
|  | **TAM 115** | 7D | 55237494 | 616620261 | 95.9 | 10.9 | Yes |
|  |  | tamGB3 | 125772 | 2440501 | 85.0 |  |  |
|  | **TAM 204** | 7D | 59994359 | 613178323 | 95.4 | 9.6 | Yes |
|  |  | tamGB3 | 142869 | 2462980 | 85.8 |  |  |
|  | **KS96WGRC40** | 7D | 21620508 | 523267761 | 81.4 | 31.8 | No |
|  |  | tamGB3 | 29908 | 1422942 | 49.6 |  |  |
|  | ᶳChrom is for chromosome contig referenced. ᶤCov stands for Coverage. Coverage is calculated by the percentage of covered bases on chromosome 2A. Jagger (JG) and Jagger Rye (JG:1R) reference genomes. With Δ meaning the change. ¹Yes and no values are confirmed by marker screening data | | | | | | |
|  |  |  |  |  |  |  |  |

| Table S8. SnpEff Variant Location. | | | | |  |  |  |  |  |  |  |  |  |  |
| --- | --- | --- | --- | --- | --- | --- | --- | --- | --- | --- | --- | --- | --- | --- |
|  |  | Unfiltered Data | | | | | | Filtered^1^ Data | | | | | | |
|  | | Chinese Spring (CS) | | Jagger (JG) | | Durum Tauschii (DT) | | Chinese Spring (CS) | | Jagger (JG) | | Durum Tauschii (DT) | |  |
| Variant Location | | Count | Percentᶤ | Count | Percent | Count | Percent | Count | Percent | Count | Percent | Count | Percent |  |
| **Downstream** | | 23,511,083 | 8.66% | 12,017,639 | 5.48% | 52,015,220 | 12.08% | 1,401,596 | 9.09% | 802,357 | 5.54% | 2,705,508 | 14.31% |  |
| **Exon** | | 3,058,152 | 1.13% | 2,014,550 | 0.92% | 4,728,957 | 1.10% | 100,797 | 0.65% | 59,982 | 0.41% | 79,380 | 0.42% |  |
| **Gene** | | 17 | 0% | 4 | 0% | 772 | 0% | 1 | 0% | 0 | 0 | 103 | 0.00% |  |
| **Intergenic** | | 215,251,821 | 79.24% | 188,878,212 | 86.10% | 263,488,367 | 61.18% | 12,115,549 | 78.55% | 12,512,975 | 86.34% | 11,402,598 | 60.32% |  |
| **Intron** | | 4,780,726 | 1.76% | 3,008,002 | 1.37% | 21,991,686 | 5.11% | 223,939 | 1.45% | 155,161 | 1.07% | 679,099 | 3.59% |  |
| **Splice Site Acceptor** | | 8,670 | 0.00% | 5,784 | 0.00% | 20,556 | 0.01% | 354 | 0.00% | 205 | 0.00% | 304 | 0.00% |  |
| **Splice Site Donor** | | 7,640 | 0.00% | 5,269 | 0.00% | 15,163 | 0.00% | 324 | 0.00% | 195 | 0.00% | 338 | 0.00% |  |
| **Splice Site Region** | | 92,963 | 0.03% | 74,602 | 0.03% | 203,786 | 0.05% | 4,487 | 0.03% | 3,658 | 0.03% | 22,008 | 0.12% |  |
| **Transcript** | | 4,278 | 0.00% | 474 | 0% | 34,278,094 | 7.96% | 75 | 0% | 71 | 0% | 1,072,967 | 5.68% |  |
| **Upstream** | | 24,291,456 | 8.94% | 12,543,400 | 5.72% | 53,382,985 | 12.39% | 1,534,087 | 9.95% | 906,946 | 6.26% | 2,929,760 | 15.50% |  |
| **UTR 3 Prime** | | 393,302 | 0.15% | 491,014 | 0.22% | 300,986 | 0.07% | 28,034 | 0.18% | 33,297 | 0.23% | 6,478 | 0.03% |  |
| **UTR 5 Prime** | | 258,903 | 0.10% | 327,627 | 0.15% | 276,037 | 0.06% | 14,363 | 0.09% | 18,257 | 0.13% | 5,843 | 0.03% |  |
| Variant Effect | | Count | Percent | Count | Percent | Count | Percent | Count | Percent | Count | Percent | Count | Percent |  |
| **High** | | 142,821 | 0.05% | 69,846 | 0.03% | 203,740 | 0.05% | 8,591 | 0.06% | 3,205 | 0.02% | 3,788 | 0.02% |  |
| **Low** | | 1,211,596 | 0.45% | 870,121 | 0.40% | 1,598,250 | 0.37% | 38,309 | 0.25% | 27,194 | 0.19% | 48,472 | 0.26% |  |
| **Moderate** | | 1,842,125 | 0.68% | 1,197,055 | 0.55% | 2,092,876 | 0.49% | 60,463 | 0.39% | 35,431 | 0.24% | 37,052 | 0.20% |  |
| **Modifier** | | 268,462,469 | 98.82% | 217,229,555 | 99.03% | 426,807,743 | 99.10% | 15,316,243 | 99.30% | 14,427,274 | 99.55% | 18,815,074 | 99.53% |  |
| Variant Changes | | Count | Percent | Count | Percent | Count | Percent | Count | Percent | Count | Percent | Count | Percent |  |
| **Missense** | | 1,848,528 | 61.21% | 1,194,005 | 59.69% | 2,083,349 | 59.06% | 59,941 | 63.11% | 34,644 | 60.17% | 35,753 | 56.88% |  |
| **Non-Sense** | | 71,773 | 2.38% | 41,467 | 2.07% | 67,435 | 1.91% | 2,157 | 2.27% | 920 | 1.60% | 710 | 1.13% |  |
| **Silent** | | 1,099,478 | 36.41% | 764,805 | 38.24% | 1,376,896 | 39.03% | 32,881 | 34.62% | 22,018 | 38.24% | 26,392 | 41.99% |  |
| **Total** | | 3,019,779 | 1 | 2,000,277 | 1 | 3,527,680 | 1 | 94,979 | 1 | 57,582 | 1 | 62,855 | 1 |  |
| ᶤThe output of snpEff statistics, displaying the number of variants discovered and the percentage of the total variants. ^1^Filtered variants are filtered for minimum missing values of 10%, maximum heterozygosity of 10%, and minor allele frequency of 5%. | | | | | | | | | | | | | | |
