# Supplementary material for: Diverse reference genomes detect variants in the US winter wheat
Source: Plant Genome. 2025 Dec 30;19(1):e70160. doi: 10.1002/tpg2.70160 (PMC12750494; doi:10.1002/tpg2.70160)

Figure S1. Phylogenetic tree of thirty evaluated lines aligned to Chinese Spring. Different coloring bars represent the research programs that released the varieties. The length of the colored section of the phylogenetic tree corresponds to the genetic distance between that line and the rest of the cohort. The bar thickness is non-informative. Individuals that cluster together are more genetically similar.

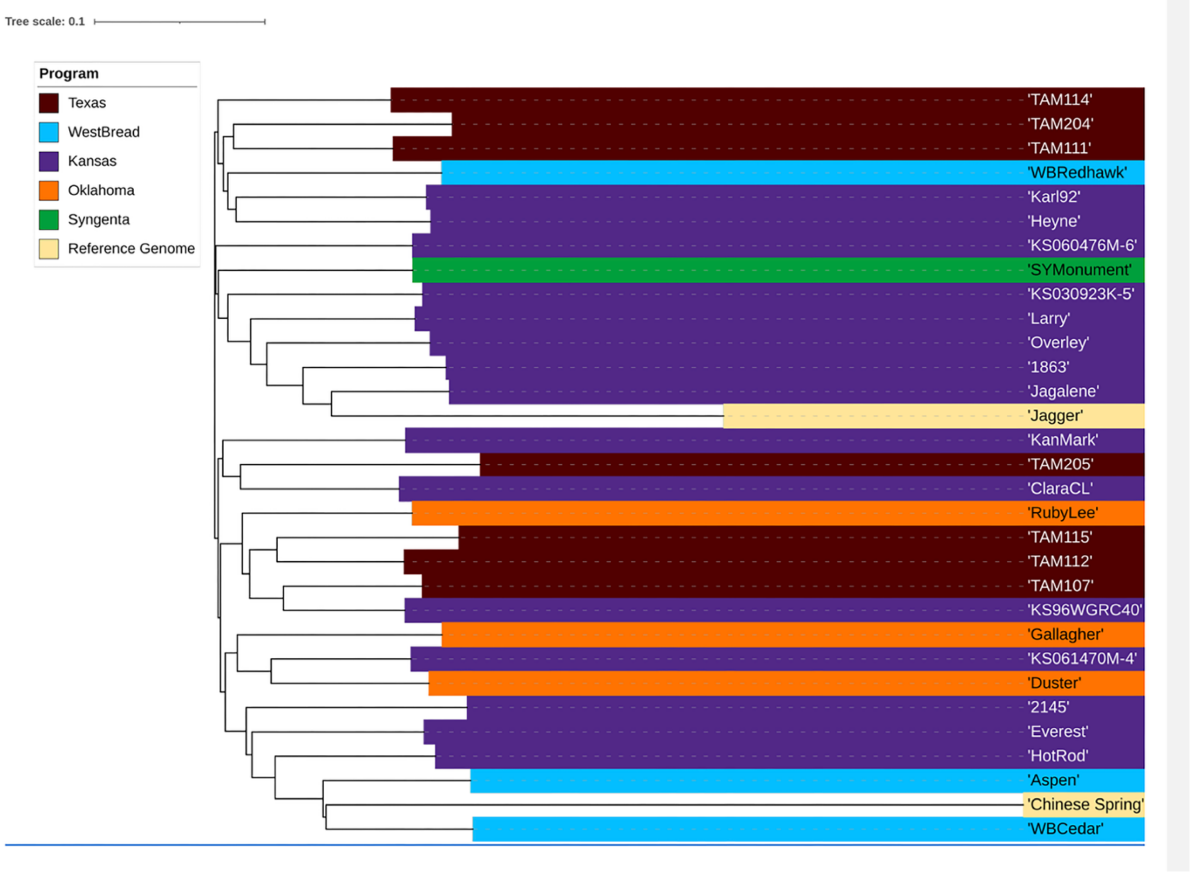

Figure S2. Distribution of alignment metrics for each Chinese Spring (CS), Durum-Tauschii (DT), and Jagger (JG). A) percentage of unmapped reads, B) duplicate aligned reads, C) percentage of reads mapped with a Phred score of 0 (MQ0), D) percentage of reads with pairs on different chromosomes, E) error rate of base mapping per reference, F) average Mapping Quality per respective reference.

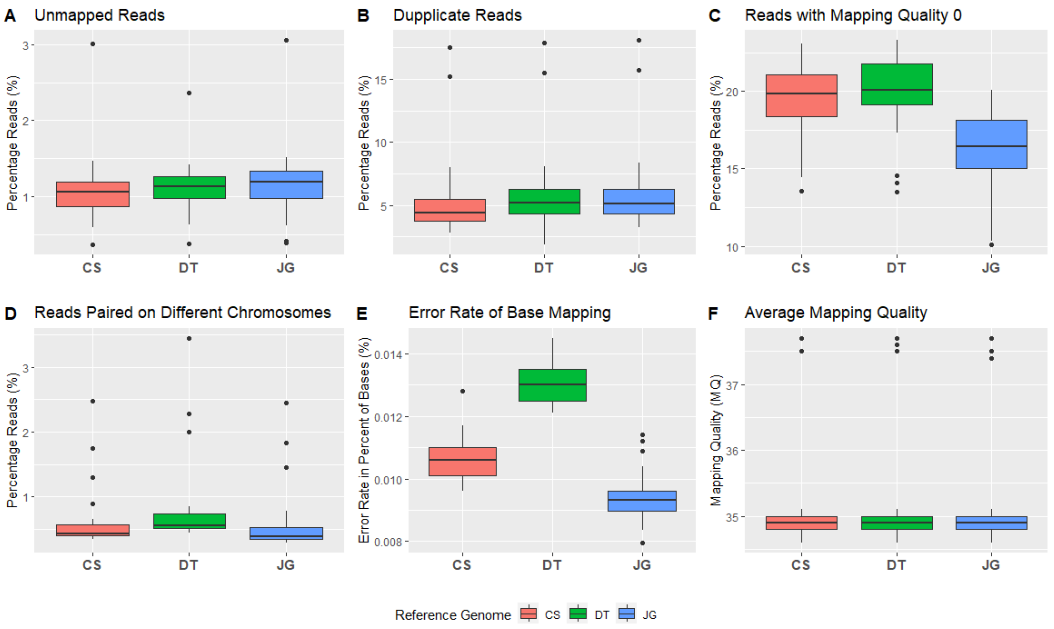

Figure S3. Coverage by chromosome. Percent coverage, calculated by number of bases covered out of the total bases of each chromosome in respects to each of the three references. Chinese Spring (CS) (red), Durum Tauschii (DT) (green), and Jagger (JG) (blue) references. These lower coverage dots correspond to particular individuals that showed a similar level of coverage across all references. In particular for chromosome 1A, cultivars Aspen, KS96WGRC40, TAM107, WBCedar and TAM 112 only showed when aligned with Jagger reference. This directly corresponds to the presence/predicted presence of the 1AL:1RS translocation, which is discussed later in the paper. Similarly, for chromosome 1B Gallagher and Larry contain the 1BL:1RS translocation, which is reflected in the low 1B alignment coverage. Lastly for chromosome 1D, cultivar KS96WGRC40 had the lowest coverage reflecting this particular individual divergence from the 1D reference chromosomes provided in this analysis.

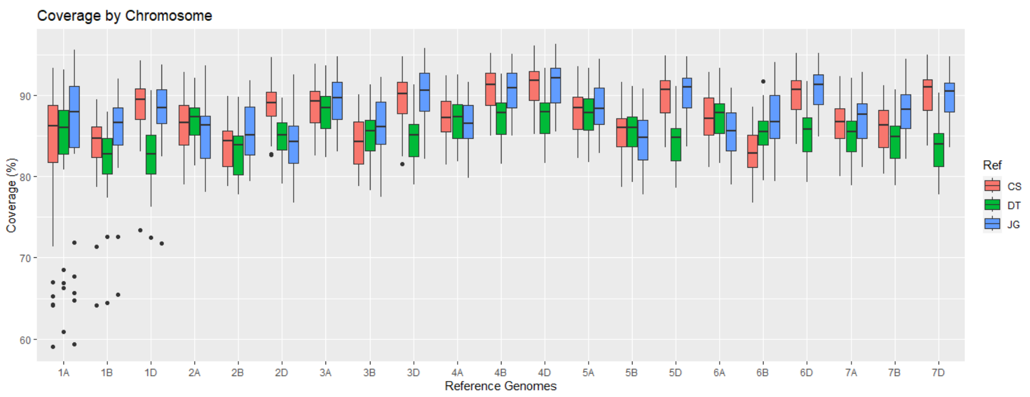

Figure S4. Alignment coverage along *tamGB3* in the number of reads per 1000bps along *tamGB3*. From left to right, Region1, Region2, and Region3 are highlighted with red boxed around the region. TAM 112, TAM 115, and TAM 204 have *Gb3*.

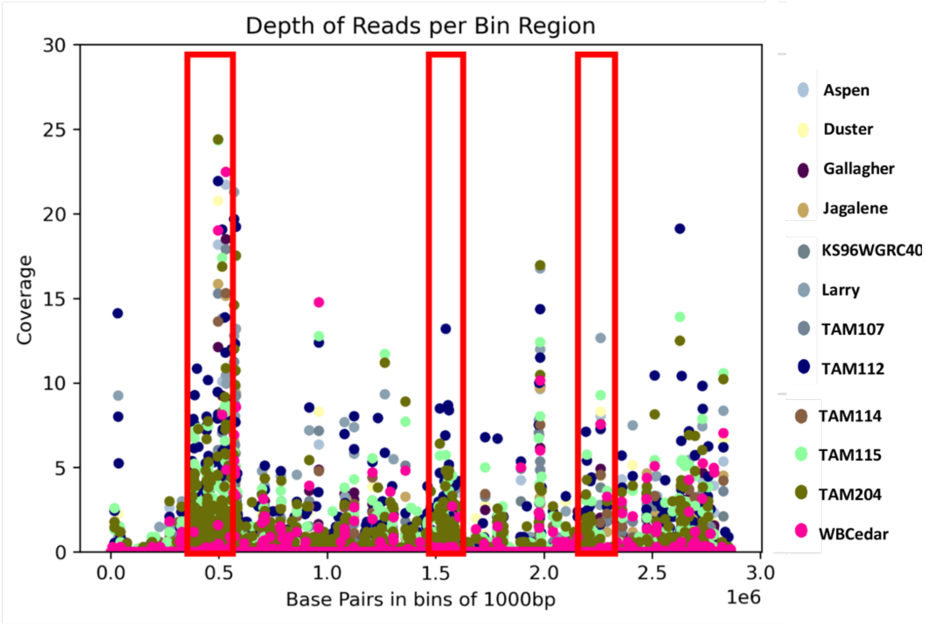

Figure S5. Allele Statistics. Distribution of allele metrics, A) Percentage of heterozygous reads on raw variant files, B) Percentage of missing alleles, C) Percentage of reference alleles in both rand filtered variant files, in reference to each reference genome Chinese Spring (CS), Durum Tauschii (DT), and Jagger (JG) using the raw variant file.

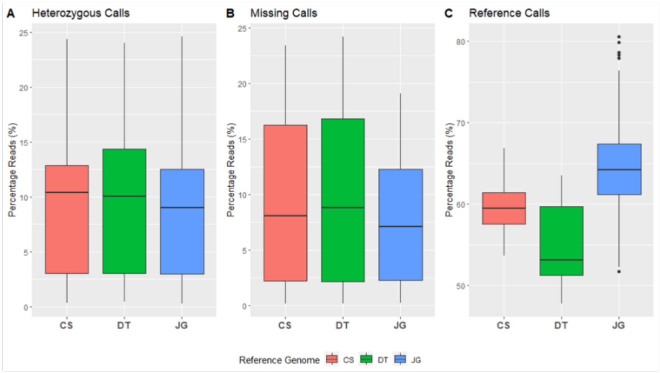

Figure S6. Gene Collinearity of reference Durum Tauschii (DT) and Chinese Spring (CS) in reference to Jagger (JG). The changes in color represent breakages in collinearity between JG and the other references. *The colors are generated at random and do not represent anything other than a break from collinearity.

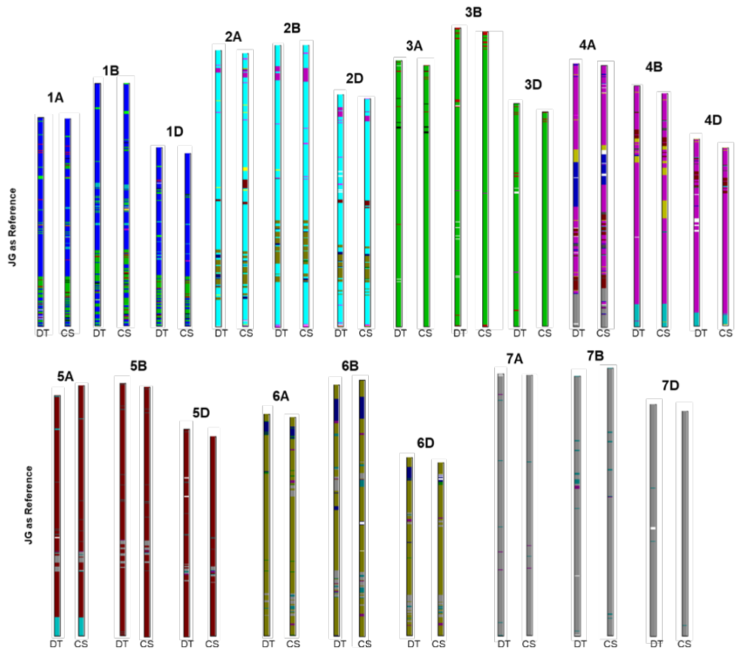

Figure S7. Pairwise synteny of chromosome 4A. a) Dot synteny plot (left) of the pairwise comparison of chromosome 4A, one can observe evidence of translocations occurring in Jagger

(JG) in comparison to Chinese Spring (CS) as well as duplication events. b) The Dual synteny plot (right) shows the linear path of collinearity, similarly, demonstrating evidence of translocations. The changes in colors represent breaks from collinear blocks, however the colors are generated at random.

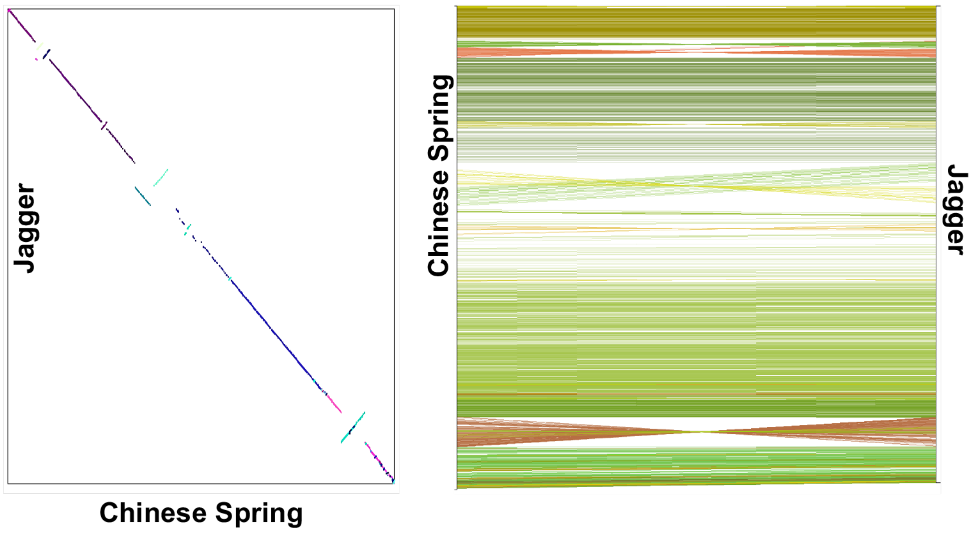

Figure S8. Alignment coverage of lines without $2N^{V}S$ (left) across Jagger chromosome 2A, and those with $2N^{V}S$ (right). The highlighted region is the ~33 Mbp region known to contain $2N^{V}S$ in Jagger.

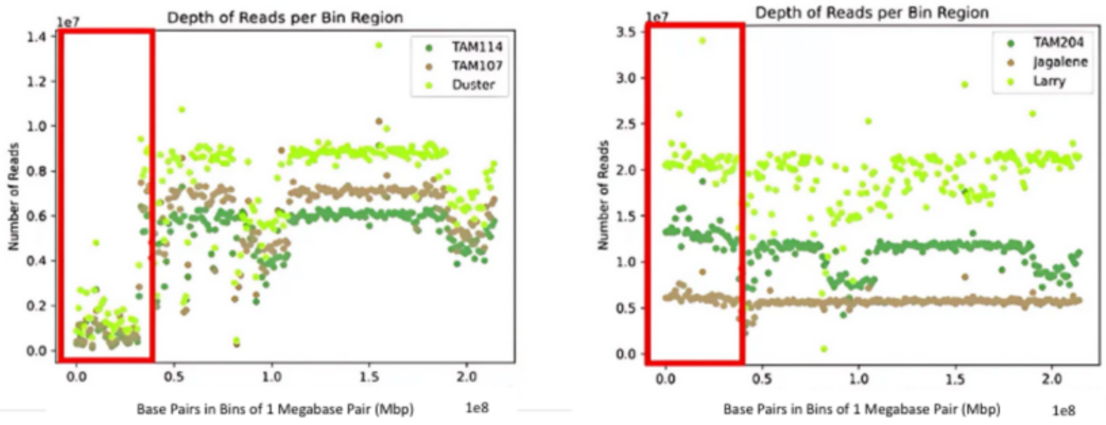

Supplement: Supplementary file 2 — Supplementary Material [file TPG2-19-e70160-s001.pdf]
